# Exploring humidity effects on polycrystalline human insulin–ligand complexes: preliminary crystallographic insights

**DOI:** 10.1107/S1600576725007484

**Published:** 2025-10-10

**Authors:** Angelos Kontarinis, Christina Papaefthymiou, Stamatina Kafetzi, Marios Konstantopoulos, Dimitris Koutoulas, Max Nanao, Gerd Schluckebier, Mathias Norrman, Natalia Dadivanyan, Detlef Beckers, Thomas Degen, Eleftheria Rosmaraki, Andrew Fitch, Irene Margiolaki

**Affiliations:** a Department of Biology, Section of Genetics, Cell Biology and Development University of Patras Patras GR-26500 Greece; b European Synchrotron Radiation Facility (ESRF) 71 Avenue des Martyrs Grenoble F-38000 France; c Therapeutic Discovery Novo Nordisk A/S Novo Nordisk Park Malov DK 2760 Denmark; d Diabetes Protein Engineering Novo Nordisk A/S Novo Nordisk Park Malov DK 2760 Denmark; e Malvern Panalytical BV, Lelyweg 1, Almelo, 7602 EA, The Netherlands; Oak Ridge National Laboratory, USA; North Carolina State University, USA

**Keywords:** *in situ*X-ray powder diffraction, XRPD, single-crystal X-ray diffraction, SCXRD, protein crystallography, relative humidity effects, human insulin complexes, pharmaceutical crystallography, structural stability, ligand binding, unit-cell variation, protein–ligand interactions

## Abstract

This study investigates the effect of relative humidity (RH) on the crystal structures of human insulin (HI) complexes with organic ligands, *m*-cresol and *m*-nitro­phenol, using *in situ*X-ray powder diffraction (XRPD) with a controlled-humidity chamber. Co-crystallization at pH 7.5 produced hexagonal microcrystals (space group *R*3) for both protein–ligand complexes. The corresponding single-crystal X-ray diffraction structures were solved: HI–*m*-cresol (PDB entry 9ibb, 1.84 Å) and HI–*m*-nitro­phenol (PDB entry 9qld, 2.55 Å). Pawley analysis of the *in situ* XRPD data revealed structural stability up to 70% RH, with no phase transitions observed. At lower humidity levels, reduced diffraction intensities indicated loss of crystallinity, which was fully restored upon rehydration to 95% RH. Notably, each complex exhibited distinct changes in unit-cell parameters during dehydration–rehydration cycles. These results highlight the critical role of controlling environmental factors in structure-based drug design and pharmaceutical manufacturing, and demonstrate how organic ligands can enhance the stability of protein crystals, offering valuable insights for pharmaceutical development.

## Abstract 2

This study explores the impact of controlled relative humidity variations on the stability and structural response of crystalline insulin complexes, combining *in situ*X-ray powder diffraction (XRPD) with single-crystal X-ray diffraction. The results reveal significant alterations in unit-cell parameters and differing stability among insulin complexes with *m*-cresol and *m*-nitro­phenol, highlighting *in situ* XRPD as a powerful tool for assessing the influence of environmental factors on the stability of pharmaceutical proteins.

## 1. Introduction

Proteins are generally stored in aqueous solutions in laboratory settings, as this environment preserves their structural integrity and functional activity. In protein crystals, individual protein molecules are arranged within a crystal structure, stabilized primarily by weak intermolecular forces such as hydrogen bonds and van der Waals interactions (Hubbard & Kamran Haider, 2010 ▸). The interstitial spaces within the structure are occupied by solvent molecules, predominantly water. Water plays a crucial role in protein crystallography by stabilizing secondary structural elements, mediating protein–protein and protein–ligand interactions, and facilitating processes such as allosteric regulation and electron transfer (Ball, 2008 ▸). Consequently, protein crystals contain a high solvent content, typically ranging from 30% to 70%, and in some cases exceeding 90% (McPherson *et al.*, 1995 ▸).

Variations in physical and chemical conditions, such as temperature, relative humidity (RH) and pH, can significantly affect the internal structure of protein crystals. Specifically, changes in RH can lead to alterations in crystal dimensions, symmetry, unit-cell parameters and the packing arrangement of constituent molecules. Protein crystals typically form under high RH conditions (95–99%), as they originate from aqueous solutions (Dobrianov *et al.*, 2001 ▸). Gradual dehydration disturbs the equilibrium between the crystal and its environment. As water is removed from solvent channels, intermolecular forces, such as hydrogen bonding and electrostatic interactions, that stabilize the crystal structure are altered, resulting in structural rearrangements (Heras & Martin, 2005 ▸).

These changes are particularly relevant for pharmaceutical development and storage, where maintaining structural integrity is essential for ensuring drug efficacy (Datta & Grant, 2004 ▸). *In situ* studies under controlled RH and temperature conditions are crucial for tracking these changes over time and optimizing stability (Dobrianov *et al.*, 2001 ▸). This study investigates the effect of RH on the crystal structures of human insulin (HI) complexes with organic ligands using *in situ*X-ray powder diffraction (XRPD). Advances in instrumentation, data collection and analysis methods (Karavassili *et al.*, 2017 ▸; Margiolaki & Wright, 2008 ▸; Margiolaki, 2019 ▸) have established XRPD as a powerful technique, not only for identifying protein structures (Margiolaki *et al.*, 2007 ▸; Spiliopoulou, Karavassili *et al.*, 2021 ▸; Karavassili *et al.*, 2020 ▸; Triandafillidis *et al.*, 2023 ▸; Fili *et al.*, 2019 ▸) and crystal polymorphs (Basso *et al.*, 2005 ▸; Collings *et al.*, 2010 ▸; Karavassili *et al.*, 2012 ▸; Spiliopoulou, Valmas *et al.*, 2021 ▸; Valmas *et al.*, 2015 ▸) but also for characterizing microcrystalline samples and providing representative structural data. Moreover, XRPD is uniquely suited for *in situ* investigations under varying environmental conditions (Athanasiadou *et al.*, 2024 ▸; Logotheti *et al.*, 2019 ▸; Trampari *et al.*, 2018 ▸), making it an ideal tool for the current study.

In this study, *in situ* XRPD data were collected using an MHC-trans chamber (Multi-sample Humidity Chamber, Anton Paar) (Zellnitz *et al.*, 2015 ▸) integrated with an X’Pert PRO diffractometer (Malvern Panalytical). The chamber is designed for polycrystalline samples and features a rotating base with eight sample positions, enabling sequential measurements under synchronized humidity and temperature conditions. While only one sample can be irradiated at a time, both sample selection and environmental parameter adjustment are fully automated via dedicated software. The MHC-chamber provides precise control of RH and temperature, with RH regulation possible between +10°C and +80°C. Full RH control (5%–95%) is achievable between +20°C and +60°C. The integrated system ensures uniform heating/cooling, dehydration/rehydration and stable, homogeneous conditions around the sample. Environmental conditions are regulated by two external control units – one for RH and one for temperature – each connected to high-precision sensors for real-time monitoring and adjustment. RH is controlled using dry air supplied by a compressor with a maximum flow rate of 250 ml min^−1^.

The protein analyzed in this study, HI, is a peptide hormone secreted by the β-cells of the islets of Langerhans. Insulin plays a critical role in regulating blood glucose levels by promoting glucose uptake in the liver, skeletal muscle and adipose tissue, preventing fluctuations that can lead to hypoglycemia or hyperglycemia (Pessin & Saltiel, 2000 ▸). Disruptions in glucose metabolism, due to partial or complete insulin deficiency, are associated with the development of diabetes mellitus (Wilcox, 2005 ▸).

**Figure 1:**
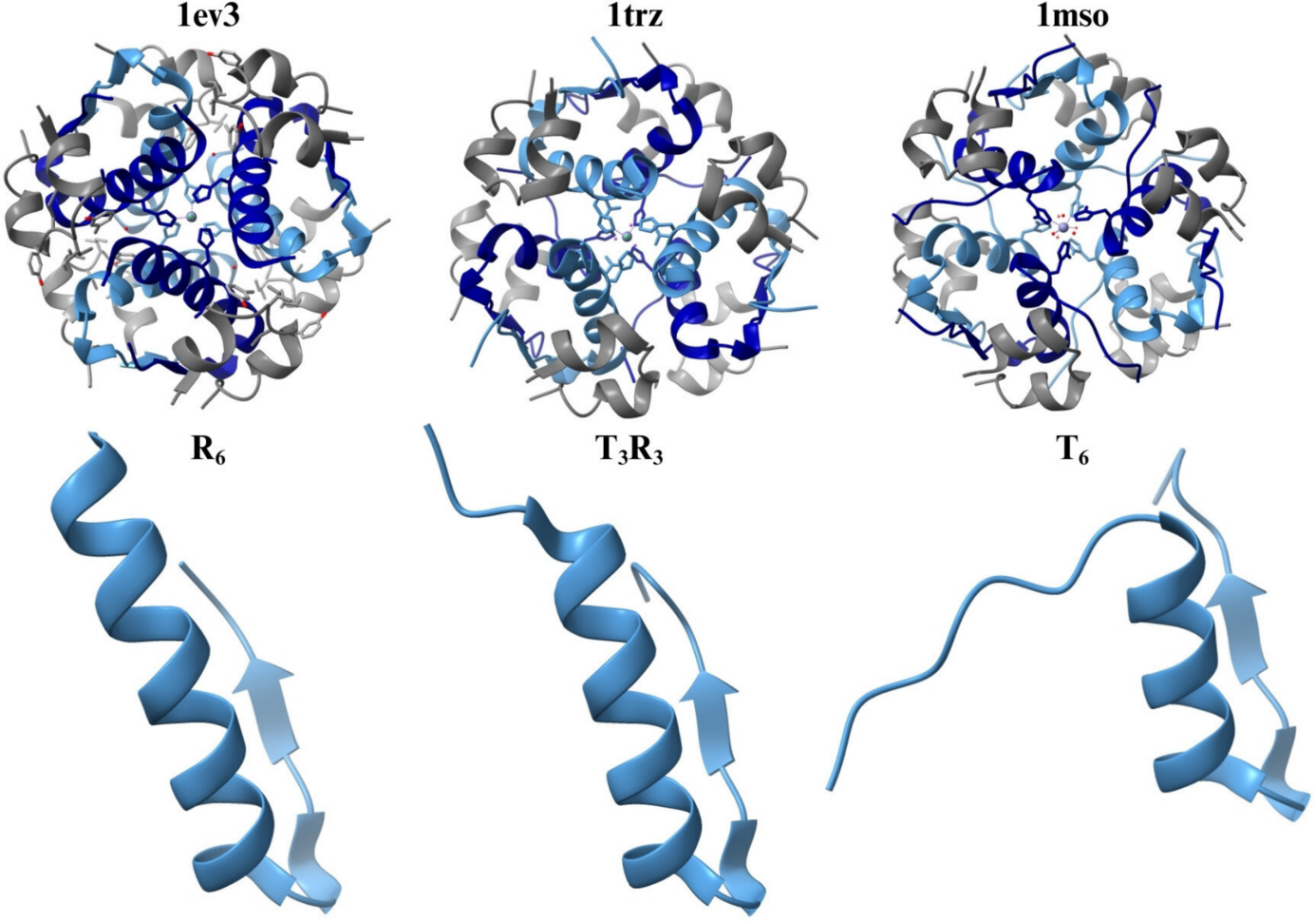
The three types of hexameric conformations of HI. Upper row, from left to right: hexamers with R_6_, T_3_R*^f^*_3_ and T_6_ conformations [PDB entries: 1ev3, 1trz (Ciszak & Smith, 1994 ▸) and 1mso (Smith *et al.*, 2003 ▸), respectively]. Lower row, from left to right: the N-terminal of chain B, illustrating differences in the conformation of the first eight residues, which result in the R, R*^f^* and T conformations.

In its active form, insulin exists as a monomer; however, after production, it is stored in secretory granules as hexameric crystals (Dunn, 2005 ▸). These hexamers can adopt three distinct configurations, T_6_, T_3_R*^f^*_3_ and R_6_ (Fig. 1 ▸), depending on the binding of organic molecules to two classes of allosteric sites: phenolic pockets that bind phenolic ligands and anion sites like HisB10-Zn^2+^ that bind monovalent anions (Dunn, 2005 ▸; Ballet *et al.*, 2010 ▸). The phase transition from T_6_ to T_3_R*^f^*_3_ can also be driven by halide concentration and pH (Reynolds *et al.*, 1988 ▸). The crystalline polymorphism of HI has been extensively studied, revealing the various ways insulin molecular polymorphs are packed in crystals (Karavassili *et al.*, 2017 ▸; Triandafillidis *et al.*, 2023 ▸, 2020 ▸). Although HI is a treatment for all types of diabetes, it is ineffective when administered orally because gastric enzymes break it down before absorption. Consequently, insulin is delivered subcutaneously in the form of microcrystals or as a combination of microcrystals and amorphous protein (Valmas *et al.*, 2015 ▸).

In this study, two phenolic ligands, *meta*-cresol (*m*-cresol) and 3-nitro­phenol (*m*-nitro­phenol), were used as co-crystallization ligands with insulin. Since these ligands exhibit antimicrobial properties, knowledge of their influence on crystallization and structure is important for insulin pharmaceutical formulations. *m*-Cresol is already widely used as a preservative in commercial insulin products, including the rapid-acting insulin analog aspart. Meanwhile, nitro­phenols, such as *m*-nitro­phenol, are primarily known for their bactericidal effects and serve as intermediates in the chemical synthesis of mesalazine, an active substance used to treat ulcerative colitis and Crohn’s disease (Karavassili *et al.*, 2020 ▸; Le *et al.*, 2021 ▸; Spain *et al.*, 2000 ▸; Valmas *et al.*, 2015 ▸). The presence of small organic compounds, such as phenolic derivatives, resorcinol or benzoic acid ligands, influences crystal formation, leading to a wide range of polymorphs. To date, more than 32 insulin crystal polymorphs have been identified (Valmas *et al.*, 2015 ▸; Karavassili *et al.*, 2017 ▸, 2012 ▸; Spiliopoulou, Valmas *et al.*, 2021 ▸; Triandafillidis *et al.*, 2020 ▸; Fili *et al.*, 2015 ▸).

## 2. Experimental

### 2.1. Materials

For the formation of HI hexamers, we used recombinant HI provided by Novo Nordisk A/S. Zinc acetate (CAS No. 5970-45-6) was procured from Sigma–Aldrich, while sodium thio­cyanate (CAS No. 540-72-7) was obtained from Fluka Chemie. The organic ligands *m*-cresol (CAS No. 108-39-4) and *m*-nitro­phenol (CAS No. 554-84-7) were procured from Merck KGaA. The reagents utilized to generate a pH buffer included sodium dihydrogen phosphate 2-hydrate (CAS No. 13472-35-0), obtained from PanReac AppliChem ITW Reagents, and di­potassium hydrogen phosphate (CAS No. 7758-11-4), purchased from Merck KGaA. Ultrapure water (UPW) obtained after desalinization, reverse osmosis and deionization with a conductivity of 0.05501 µS cm^−1^ at 25 °C was used for all experiments.

### 2.2. Crystallization

The crystallization of HI was carried out using the salting-out method (Hofmeister, 1888 ▸) in batch mode, as described previously (Valmas *et al.*, 2015 ▸). To ensure the reproducibility of our results, we conducted two crystallization series for the ligand *m*-cresol (referred to as mcre1 and mcre2) and four crystallization series for *m*-nitro­phenol (mnit1, mnit2, mnit3 and mnit4), following identical protocols for both ligands. Each series comprises six samples, and approximately 300 µl of polycrystalline material from all six samples was required for loading onto thin Kapton foil holders.

For the preparation of six samples of the HI–*m*-cresol complex (HI–mcre), a stock protein solution was made by dissolving 57.2 mg of freeze-dried HI in 3 ml of UPW, along with 348 µl of a 10 m*M* zinc acetate solution. This resulted in a protein concentration of 19.07 mg ml^−1^. To this solution, 80.4 µl of a 25% *v*/*vm*-cresol solution, diluted in ethanol, was added to create the protein–ligand mixture. After 5 min of incubation, 40 µl of a 1 *M* sodium thio­cyanate solution was added. A pH 7.50 buffer was prepared by mixing 2 *M* stock solutions of NaH_2_PO_4_·2H_2_O and K_2_HPO_4_. For each sample, 0.5 ml of the protein mixture and 125 µl of the pH-buffer mixture were combined in a 2 ml Eppendorf tube. This resulted in a final protein concentration of 13.14 mg ml^−1^ and an *m*-cresol concentration of 0.51% *v*/*v*.

**Figure 2:**
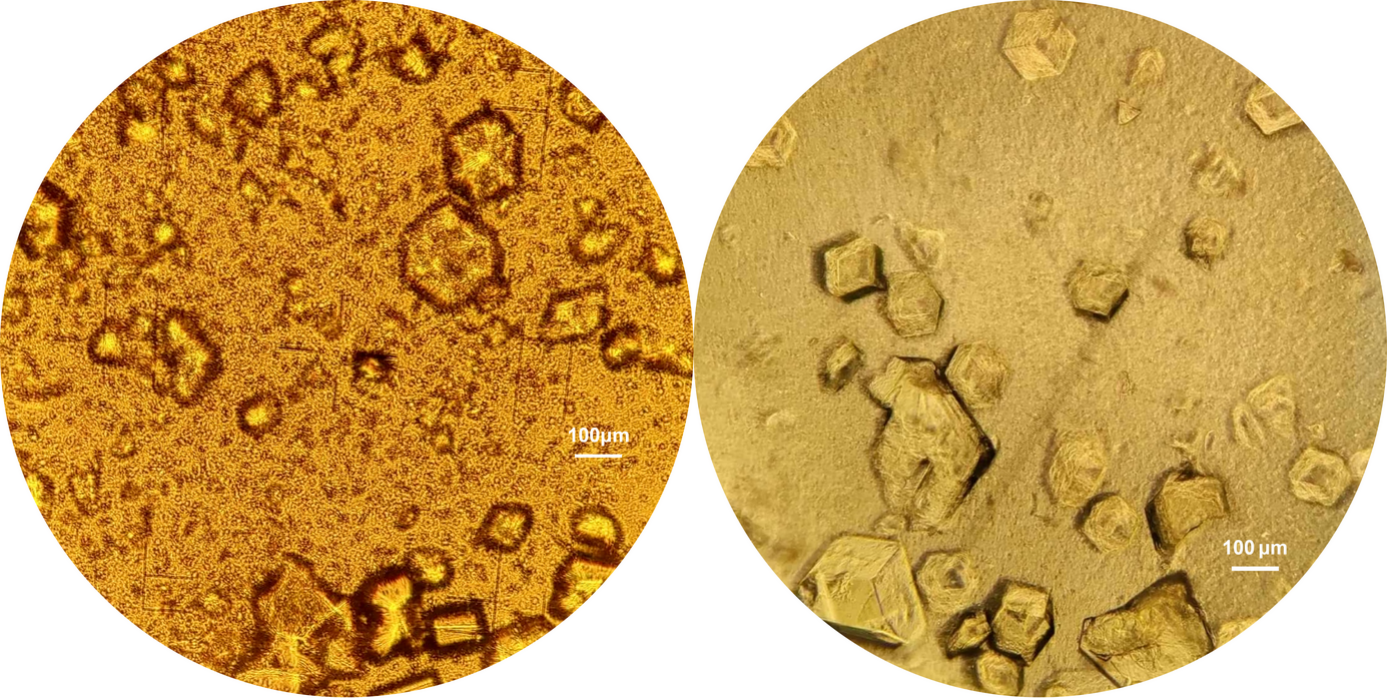
Polycrystalline samples of HI–*m*-cresol (left) and HI–*m*-nitro­phenol (right) hexagonal complexes.

For the preparation of the HI–*m*-nitro­phenol complex (HI–mnit), the procedure for each of the four series was as follows: 48.83 mg of freeze-dried HI was dissolved in 2.57 ml of UPW, before the addition of 295.7 µl of a 10 m*M* zinc acetate solution and 153.3 µl of a 1 *Mm*-nitro­phenol solution dissolved in dimethyl sulfoxide (DMSO). After 5 min, 38.6 µl of a 1 *M* sodium thio­cyanate solution was added to the mixture. The pH 7.50 buffer was prepared using NaH_2_PO_4_·2H_2_O and K_2_HPO_4_. For each sample, 0.5 ml of the protein–ligand mixture and 125 µl of the pH-buffer mixture were combined. This resulted in a final protein concentration of 12.78 mg ml^−1^ and an *m*-nitro­phenol concentration of 40.06 m*M* (Table 1 ▸). The samples (Fig. 2 ▸) were left to crystallize at room temperature (298 K) for approximately 48 h, yielding 50 ml of polycrystalline material. *In situ* XRPD data were collected within 20 days of the crystallization experiments.

Each series comprised six identical samples, hence the decision not to assign individual names. Instead, each code of the series consists of the four letters representing each ligand (mcre, mnit), followed by a number 1 or 2 indicating whether the series was conducted first or second.

Only a slight change in the pH of the sample was observed over time. The term ‘starting pH’ denotes the pH of the crystallization buffer before mixing with the protein mixture, whereas ‘final pH’ indicates the pH of the sample before *in situ* XRPD experiments. The ‘final pH’ values were 7.60 and 7.55 for samples mcre1 and mcre2, respectively, and 7.74, 7.66, 7.62 and 7.67 for samples mnit1, mnit2, mnit3 and mnit4, respectively.

### 2.3. XRPD experiments: data collection utilizing synchrotron and laboratory sources

XRPD data collection was conducted using multiple sources to take advantage of their complementary features and ensure the reproducibility of the results. Polycrystalline samples of HI complexed with *m*-cresol and *m*-nitro­phenol were first measured using an in-house diffractometer (X’Pert Pro, Malvern Panalytical). To obtain data with enhanced angular resolution, measurements were then performed at the high-resolution powder diffraction beamline ID22 (Fitch *et al.*, 2023 ▸) at the European Synchrotron Radiation Facility (ESRF) in Grenoble. In addition, *in situ* XRPD data were collected using the MHC-trans humidity chamber installed in the laboratory diffractometer.

Samples were loaded into 1.0 mm inner diameter borosilicate glass capillaries. To achieve dense crystal packing in front of the incident beam, the capillaries were centrifuged, and excess mother liquor was removed. The capillaries were sealed with silicon vacuum grease to prevent solvent evaporation and were mounted on the goniometer head of a translating capillary spinner.

Laboratory data were collected using Debye–Scherrer geometry with Cu *K*α radiation [λ = 1.540585 (3) Å] (Hölzer *et al.*, 1997 ▸) at room temperature. A focusing X-ray mirror was paired with a 1/2° anti-scatter slit, a 0.04 rad Soller slit, a 10 mm mask and a 1/2° divergence slit on the incident-beam side. On the diffracted-beam side, a 0.04 rad Soller slit and a PIXcel1D detector with anti-scatter shielding were used. Two-theta (2θ) scans were initiated from 0°, with a beamstop mounted on the anti-scatter device of the X-ray mirror to protect the detector. Each sample was measured repeatedly at a single capillary position in a 2θ range of 0° to 30°. Each scan lasted approximately 30 min, and a total of 25 scans were conducted. The scans were summed to improve counting statistics and signal-to-noise ratio, with no radiation damage observed after 12.5 h of measurements.

Synchrotron data were collected at the ESRF using a multi-crystal analyzer detector [λ = 1.30079 (2) Å, beam size 1 × 1 mm]. The capillaries were rotated at 1031 rev min^−1^ to ensure sufficient powder averaging. Periodic axial translations were performed to expose fresh sections of the sample, unaffected by radiation damage. Approximately ten scans were collected per sample at room temperature. Due to the large diameter of the capillaries, flash-cooling was not possible, and cryocooling was avoided to prevent peak broadening, ice formation or potential phase transitions (Jenner *et al.*, 2007 ▸; Margiolaki, 2019 ▸). Identical scans collected from fresh sample sections were summed to improve counting statistics without compromising data quality (Margiolaki *et al.*, 2013 ▸; Margiolaki & Wright, 2008 ▸).

### 2.4. Single-crystal data collection

Following polymorph identification via XRPD, single-crystal X-ray diffraction (SCXRD) data were collected at the ESRF’s ID23-2 beamline (Nanao *et al.*, 2022 ▸) in Grenoble. Single crystals were isolated from the precipitates of batch samples, soaked in a cryo-protectant solution containing 30% glycerol and subsequently frozen in liquid nitro­gen. Data collection was performed at 100 K using a monochromatic X-ray wavelength of λ = 0.8731 Å, a beam size of 4 × 4 µm and a flux of 2.33 × 10^11^ photons s^−1^. On the diffracted-beam side there was a Si(111) monochromator and a Dectris Eiger X 9M detector. Data processing was carried out using *AutoPROC* (Vonrhein *et al.*, 2011 ▸). The structures were solved using *Phaser* (McCoy *et al.*, 2007 ▸), with the HI structure (PDB entry 1ev3) (Smith *et al.*, 2000 ▸) serving as the search model in both cases. The structure of the HI–mcre complex was refined with *REFMAC5* (Murshudov *et al.*, 2011 ▸), while the HI–mnit complex was refined using *phenix.refine* (Afonine *et al.*, 2012 ▸; Liebschner *et al.*, 2019 ▸). Both structural models were built in *Coot* (Emsley *et al.*, 2010 ▸).

### 2.5. *In situ* XRPD data collection

*In situ* XRPD data were collected under controlled RH variations using the laboratory X’Pert Pro diffractometer (Malvern Panalytical) equipped with the transmission temperature–humidity chamber (MHC-trans, Anton Paar). The incident beam was focused with an elliptical X-ray mirror [Cu radiation, λ = 1.540585 (3) Å], coupled with a 1/4° divergence slit (∼1 mm thickness), 0.04 rad Soller slits and a 1/4° anti-scatter slit. On the diffracted-beam side, a PIXcel1D detector was used with a 7.5 mm anti-scatter slit and a 0.04 rad Soller slit. The detector operated in 1D data collection mode, with 2θ scans performed in the range of 2–30°.

Protein crystalline precipitates stored in Eppendorf tubes were centrifuged, and the excess mother liquor was discarded. The remaining precipitate was collected and loaded onto thin Kapton foil holders (125 µm thick, 12 mm in diameter) to minimize background contributions. Each sample holder was placed in a multiple-position sample holder within the humidity chamber.

To investigate the structural response of protein crystals to changes in RH, we conducted two experimental series for the HI–*m*-cresol complex, involving six measurement cycles of XRPD data collection, and four series for the HI–*m*-nitro­phenol complex, comprising eight measurement cycles (Table 2 ▸). Each cycle began at an initial RH of 95% and was performed at a constant temperature of 294.15 K. The RH variation measurement programs involved incremental steps of 5% (or 2%, depending on the cycle), starting from the initial RH, decreasing to a minimum value, and then increasing back to the initial value. After each transition, the system was allowed to equilibrate for 45, 60 or 90 min before data collection. For each step, ten XRPD patterns were recorded, with each scan lasting 10 min and the detector moving at a rate of 0.00656° per second. The number of XRPD scans per cycle was optimized to capture structural changes associated with varying RH conditions.

For the mcre1 sample, measurement cycles were conducted across a humidity range of 95% to 75% RH. The mcre2 sample included additional humidity levels of 73% and 70% RH to assess the endurance of the crystallites under lower-humidity conditions. To enhance sample equilibration at each step, the waiting time between RH transitions was extended to 90 and 60 min for mcre2, compared with 45 min in the mcre1 cycles. For mnit1 and mnit2, identical measurement cycles were conducted to assess repeatability and stability down to 70% RH, with a 90 min waiting time at each step. For subsequent cycles with mnit3 and mnit4, the waiting time was reduced to 60 min to minimize sample exposure while extending the RH range. In the final three cycles, a 5% RH transition step was introduced, lowering the final humidity level to 50% to assess the sample’s resilience under very low RH conditions. In each cycle, only one sample was measured in the MHC-trans humidity chamber, except for cycle 7 with mnit3 and cycle 8 with mnit4, where both samples were measured simultaneously following the same dehydration–rehydration program.

Following data collection, all synchrotron and laboratory XRPD patterns were analyzed using the Pawley method (Pawley, 1981 ▸) implemented in the *HighScore Plus* crystallographic package (Degen *et al.*, 2014 ▸). Pawley fits allowed us to accurately determine the unit-cell parameters and characterize peak shapes and background coefficients, providing valuable insights into the structural response of the protein crystals to RH variation. To analyze the *in situ* XRPD data at each RH level, the individual scans were not analyzed separately. Instead, all scans at the same RH level were extensively compared, and those showing no peak shifts or single-crystal peaks (spikes) were summed to generate an overall pattern for each RH level.

## 3. Results

### 3.1. R_6_ conformation in rhombohedral phase *R*3

HI co-crystallized with *m*-cresol and *m*-nitro­phenol at a pH of 7.50, resulting in the formation of a rhombohedral symmetry (space group *R*3). For the HI–*m*-cresol complex, this polymorph was previously reported by Smith *et al.* (2000 ▸) (PDB entry 1ev3), exhibiting lattice dimensions of *a* = 78.866 and *c* = 39.465 Å. These dimensions correspond to the R_6_ hexameric conformation associated with the binding of *m*-cresol, diluted in acetone, to the phenolic pockets of insulin.

**Figure 3:**
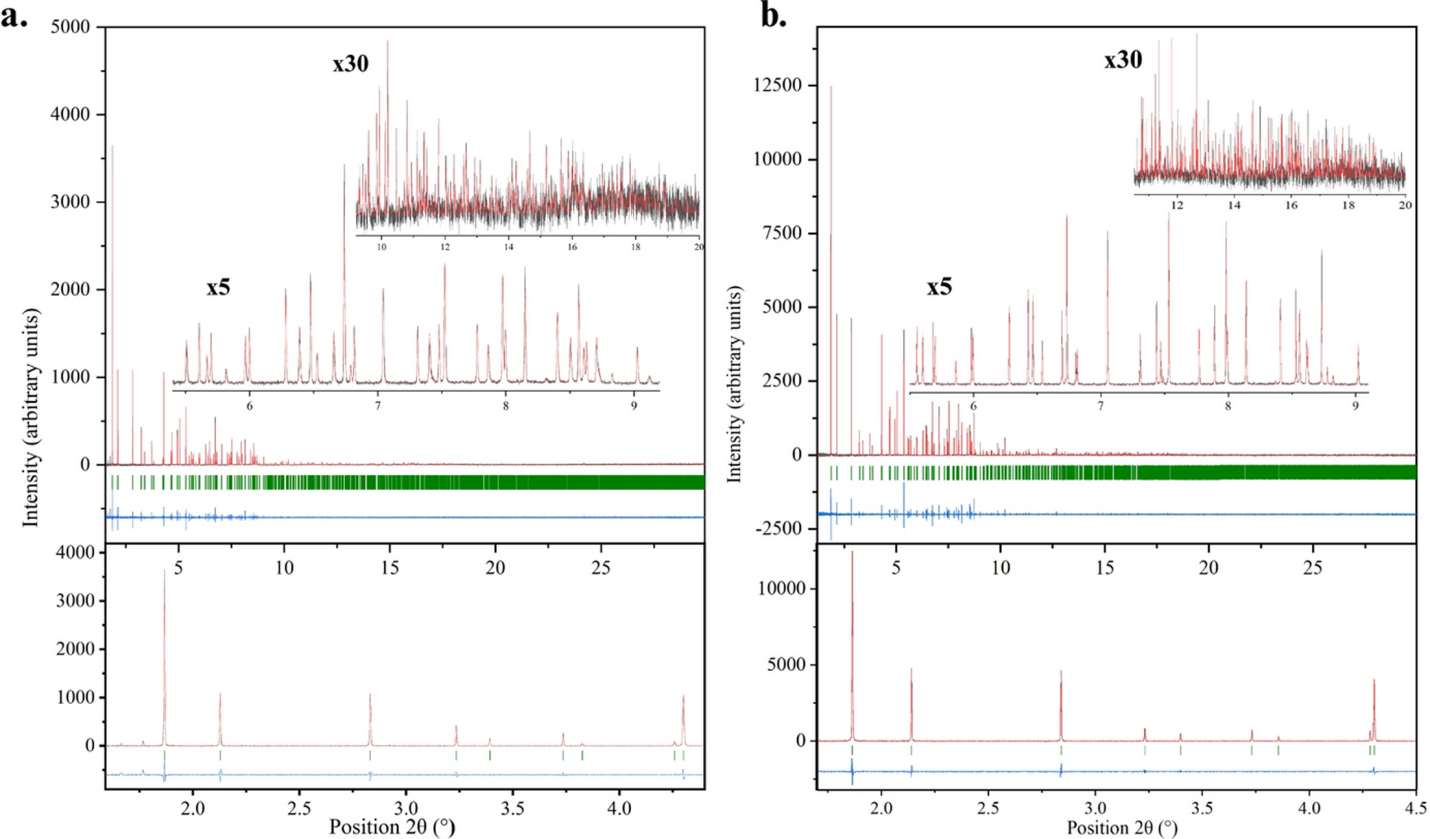
Pawley fits of synchrotron XRPD data collected for the HI complexes with *m*-cresol (*a*) and *m*-nitro­phenol (*b*) on ID22 at ESRF [λ = 1.30079 (2) Å, room temperature]. For both plots, the upper panels show the experimental data (black), calculated pattern (red) and difference profile (blue), with green vertical bars marking Bragg reflections corresponding to the rhombohedral space group *R*3 of each complex. The data were scaled by factors of 5 and 30 to emphasize the enhanced *d*-spacing resolution. The lower panels magnify the 2θ range from 1.6° to 4.5°, highlighting angular resolution with the background intensity subtracted for clarity. (*a*) HI–*m*-cresol complex with lattice parameters *a* = 79.7753 (1), *c* = 40.6005 (2) Å, with goodness of fit χ^2^ = 1.33 and *R*_wp_ = 5.97%. (*b*) HI–*m*-nitro­phenol complex with lattice parameters *a* = 79.8633 (3), *c* = 40.2489 (1) Å, with goodness of fit χ^2^ = 1.40 and *R*_wp_ = 7.51%.

High-resolution XRPD measurements conducted at the ID22 beamline (ESRF) confirmed the presence of the same rhombohedral polymorph for both HI complexes. For the HI–*m*-cresol complex, the lattice dimensions were determined to be *a* = 79.7753 (1) and *c* = 40.6005 (2) Å, with goodness of fit χ^2^ = 1.33 and *R*_wp_ = 5.97%. Similarly, for the HI–*m*-nitro­phenol complex, the lattice dimensions were *a* = 79.8633 (3) and *c* = 40.2489 (1) Å, with goodness of fit χ^2^ = 1.40 and *R*_wp_ = 7.51% (Fig. 3 ▸).

Laboratory XRPD data collected via capillary measurements using the X’Pert Pro diffractometer (Malvern Panalytical) further confirmed the presence of the rhombohedral polymorph. Pawley analysis for mcre1 yielded lattice dimensions of *a* = 80.009 (4) and *c* = 40.770 (2) Å with goodness of fit χ^2^ = 2.14 and *R*_wp_ = 0.88%. Similarly, for mnit1, the lattice dimensions were determined as *a* = 80.028 (4) and *c* = 40.325 (2) Å with goodness of fit χ^2^ = 1.19 and *R*_wp_ = 0.99%.

#### 3.1.1. Crystal structure of rhombohedral R_6_ HI complexed with *m*-cresol

**Figure 4:**
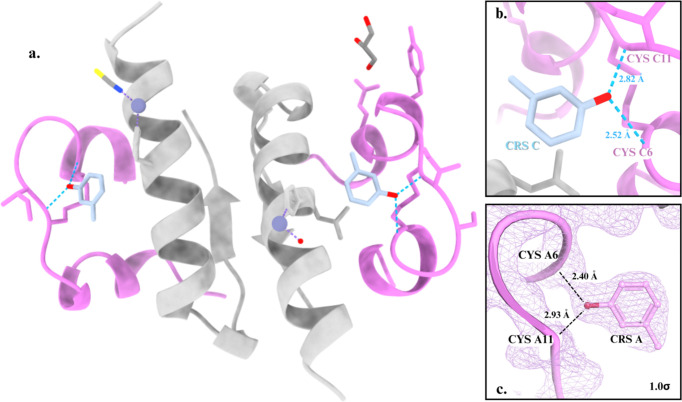
(*a*) The HI dimer complexed with *m*-cresol (PDB entry 9ibb), consisting of two insulin monomers, each bound to one *m*-cresol molecule and a zinc ion. (*b*) The phenolic pocket, where an *m*-cresol molecule forms hydrogen bonds with Cys6 and Cys11. (*c*) The 2*F*_o_ − *F*_c_ electron-density map contoured at 1.0σ, highlighting the ligand density within the binding site.

In agreement with the XRPD results, SCXRD data with a resolution of 1.84 Å from crystals grown at pH 7.5 of mcre1 samples confirmed the rhombohedral *R*3 crystalline symmetry of the HI–*m*-cresol complex, with unit-cell parameters *a* = 78.684 and *c* = 39.883 Å (Table 3 ▸). Molecular replacement solved the structure of the insulin complex with an asymmetric unit consisting of one HI dimer and a crystal solvent content of 39.93%. Structure refinement was completed with *R*_work_ = 17.791%, *R*_free_ = 22.290% and 0% Ramachandran outliers. Ligand occupancy was set to 1.0 and not refined, as the electron density was well defined and consistent with full occupancy. The dimer adopts the R_6_ molecular conformation, with each monomer containing a zinc ion coordinated to the HisB10 residue. In one monomer, the zinc ion is further coordinated by a thio­cyanate molecule, while in the other monomer it is coordinated by a water molecule. Each monomer binds one co-crystallized *m*-cresol molecule in the phenolic binding pocket, where its hydroxyl group forms hydrogen bonds with the carbonyl oxygen of CysA6 and the amide group of Cys11 (Fig. 4 ▸). Restrained refinement using non-crystallographic symmetry (NCS) was applied, and electron-density maps identified one glycerol molecule from the cryo-solution, along with water molecules interacting with the structure. The N-terminal PheB1 residue was not modeled in chain B due to the absence of electron density, and, similarly, the C-terminal residues LysB29 and ThrB30 in chains B and D were not modeled. Atomic coordinates and structure factors have been deposited in the Protein Data Bank (PDB entry 9ibb).

#### 3.1.2. Crystal structure of rhombohedral R_6_ HI complexed with *m*-nitro­phenol

**Figure 5:**
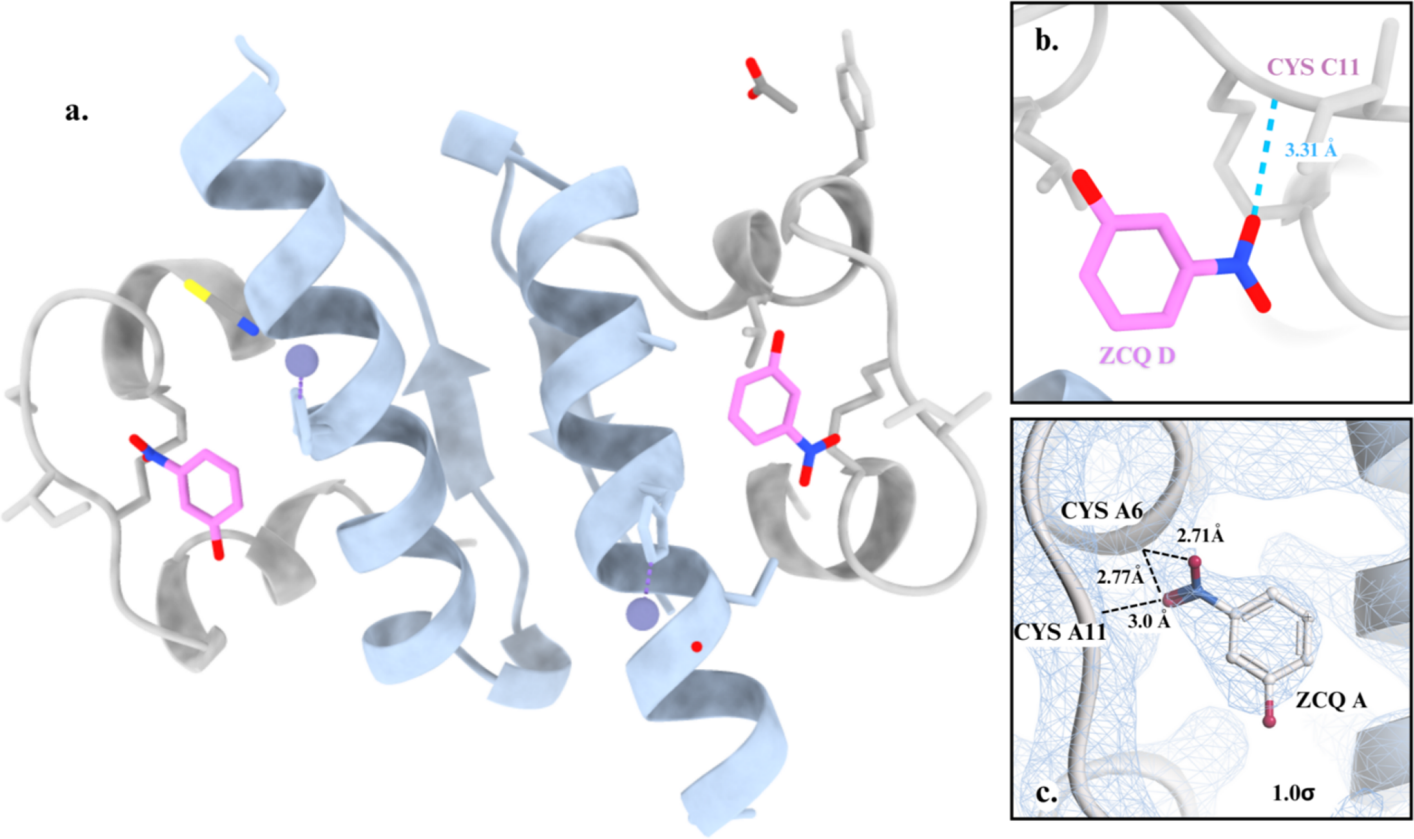
(*a*) The HI dimer complexed with *m*-nitro­phenol (PDB entry 9qld), containing two *m*-nitro­phenol molecules and two zinc ions. (*b*) One of the two phenolic pockets, where a ligand molecule forms a hydrogen bond with Cys11. (*c*) The 2*F*_o_ − *F*_c_ electron-density map contoured at 1.0σ, illustrating the density of the ligand weakly interacting with the binding site.

The SCXRD data collected from the mnit1 sample with a resolution of 2.55 Å confirmed the presence of a rhombohedral complex, consistent with the XRPD indexing results. Molecular replacement confirmed the *R*3 crystalline symmetry, with unit-cell parameters *a* = 78.932 and *c* = 39.197 Å (Table 3 ▸) and an asymmetric unit containing one HI dimer with a solvent content of 39.26%. Structure refinement was completed with *R*_work_ = 19.06%, *R*_free_ = 24.03% and 0% Ramachandran outliers. Ligand occupancy was fixed at 1.0 during refinement in *Phenix*, on the basis of clear electron density indicating full occupancy. Similar to the HI–mcre structure, the dimer adopts the R_6_ molecular conformation and exhibits the same interaction with zinc. The binding sites in this complex, however, show distinct binding patterns at the phenolic sites. In one site, the nitro group of *m*-nitro­phenol forms a hydrogen bond with Cys11, while in the other site the ligand does not engage in hydrogen bonding but rather forms only weak non-covalent interactions (Fig. 5 ▸). During restrained refinement, NCS restraints were applied, and electron-density maps revealed the presence of one acetate molecule from the crystallization solution. The C-terminal residues LysB29 and ThrB30 in chains B and D were not modeled due to the absence of electron density.

### 3.2. The effect of humidity variation on HI and *m*-cresol crystals

**Figure 6:**
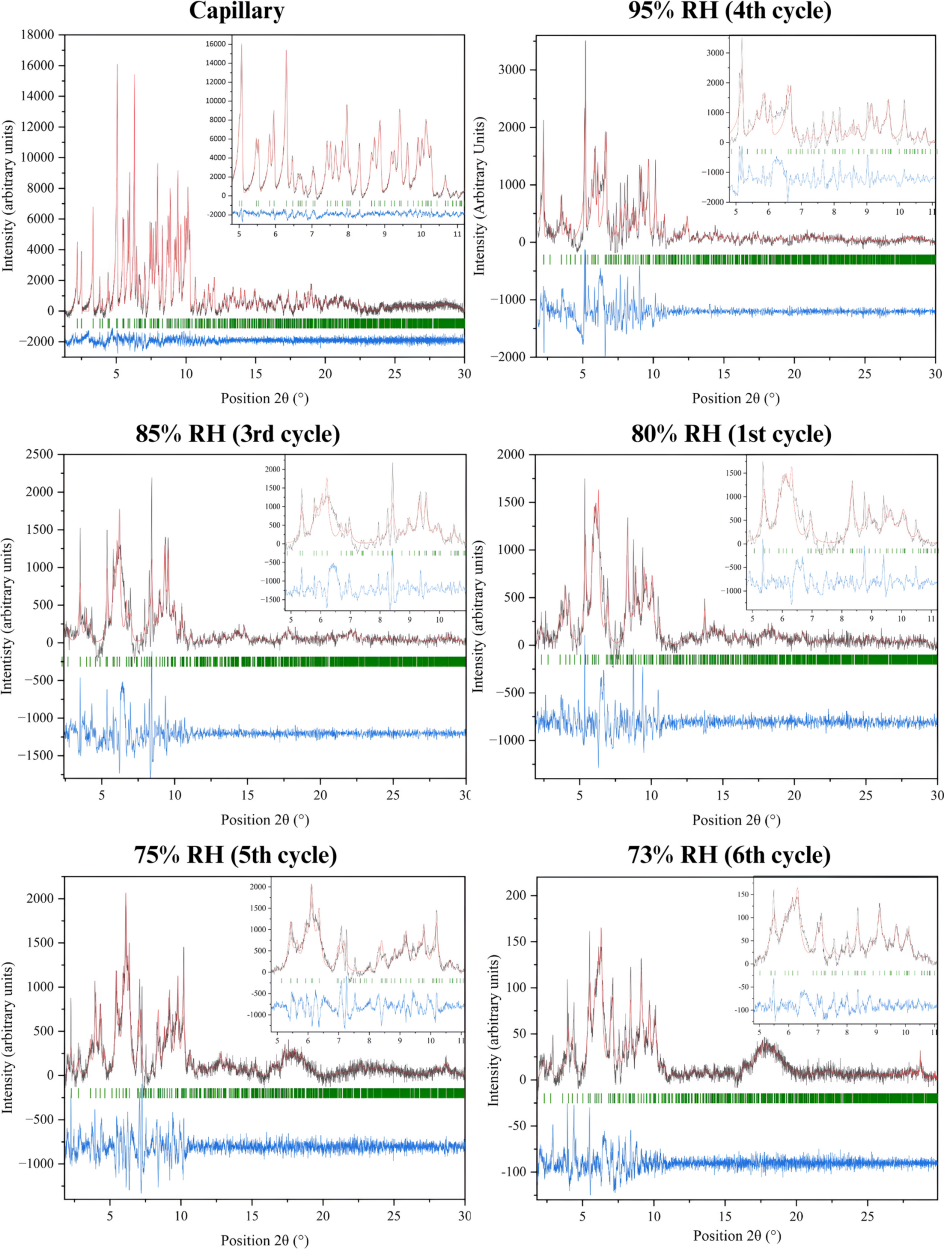
Pawley fits of XRPD data obtained with the laboratory X’Pert Pro diffractometer for the HI–*m*-cresol complex at ambient (capillary) and non-ambient conditions. In all plots, the black line represents the experimental data, the red line the calculated pattern and the blue line the difference profile, while the Bragg reflections corresponding to the rhombohedral space group *R*3 are represented by green vertical bars. The insets magnify the 2θ range from 5° to 11° corresponding to high-intensity Bragg peaks.

The gradual dehydration and subsequent rehydration of the mcre1 and mcre2 samples induced significant changes in their unit-cell parameters (see the supporting information), as shown by Pawley analysis (Fig. 6 ▸). Diffraction signals from the polycrystalline samples were detectable at humidity levels ranging from 95% to 75% RH. However, at 70% RH, the signal weakened considerably, and patterns collected during rehydration from 70% to 95% RH exhibited only faint Bragg peaks.

**Figure 7:**
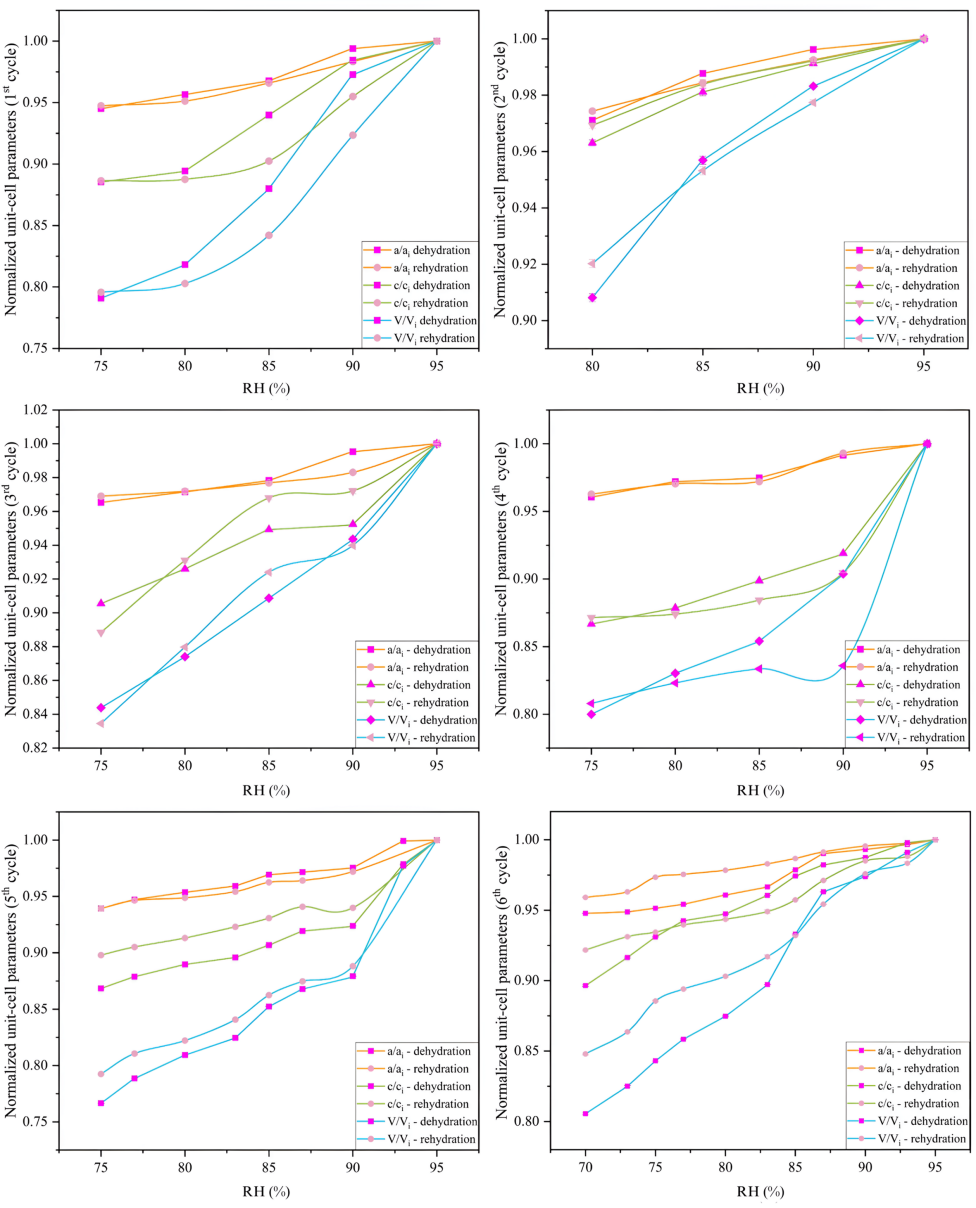
Normalized variation of unit-cell parameters *a*/*a*_i_, *c*/*c*_i_ and volume *V*/*V*_i_ as a function of RH for the six dehydration and rehydration cycles of the HI–*m*-cresol complex. The normalization was calculated relative to the initial unit-cell parameter values at 95% RH for the dehydration and rehydration process of each cycle. The plots highlight the hysteresis in unit-cell parameter variation, with distinct differences observed between dehydration and rehydration phases, indicating the structural response of the crystals to environmental changes.

**Figure 8:**
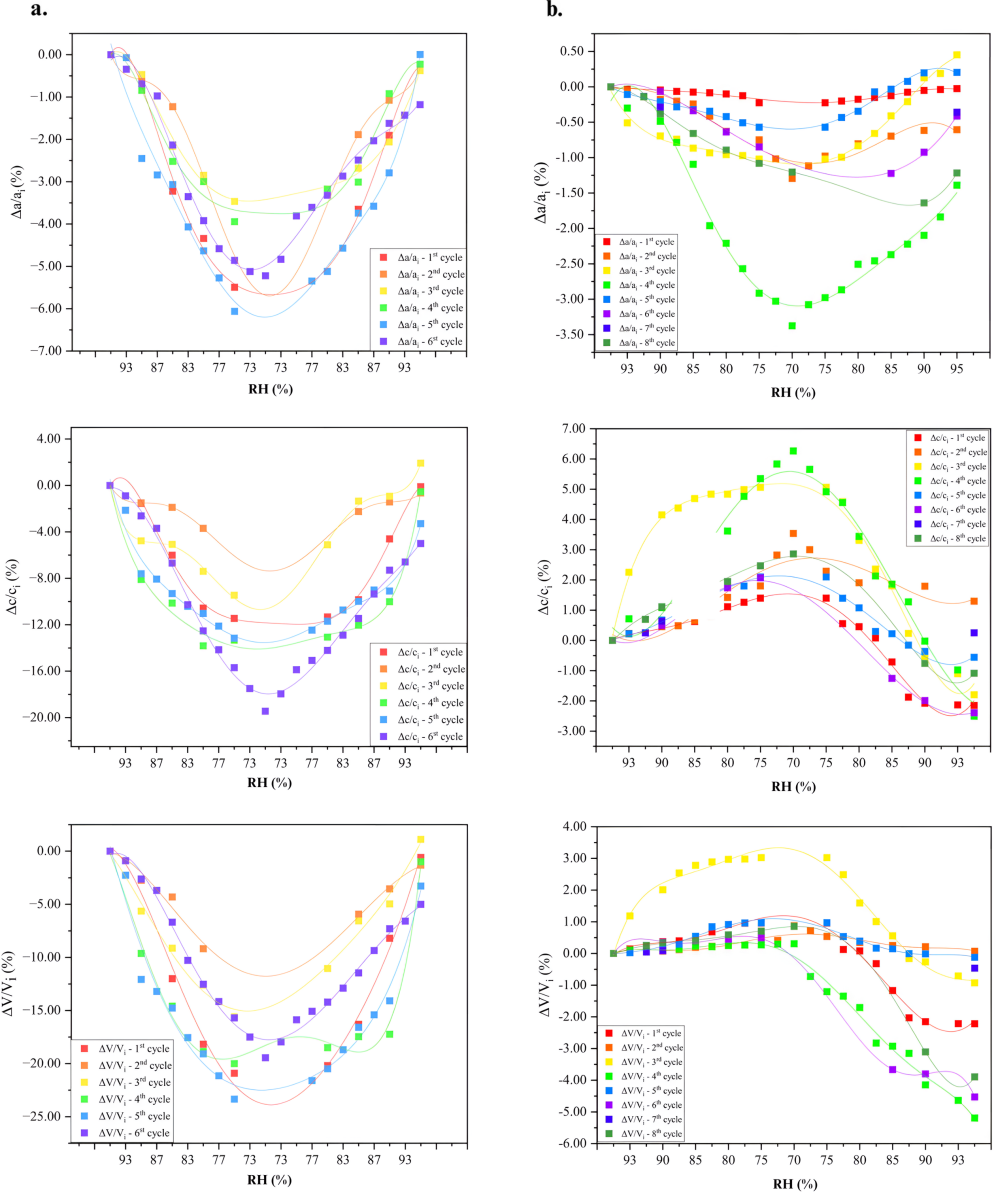
Scatter plots illustrating the delta variations in unit-cell parameters and volume, Δ*a*/*a*_i_, Δ*c*/*c*_i_ and Δ*V*/*V*_i_, for the HI complexes with *m*-cresol (*a*) and *m*-nitro­phenol (*b*). Polynomial fit lines are included as visual guides to highlight trends across cycles.

During dehydration to 75% RH, both the *a* and *c* axes of the unit cell underwent notable reductions (Fig. 7 ▸), calculated as Δ(*x*_f_ − *x*_i_)/*x*_i_ % between the initial (*x*_i_) and final (*x*_f_) values. For the mcre1 sample, during the first cycle, the *a* and *c* axes decreased by 5.49% and 11.45%, respectively, while in the third cycle, the reductions were 3.47% and 9.45%, respectively. For the mcre2 sample, during the fifth cycle, dehydration to 75% RH caused a 6.06% decrease in *a* and a 13.16% decrease in *c*. In the sixth cycle, the decreases were 5.22% and 10.35%, respectively (Fig. 8 ▸).

**Figure 9:**
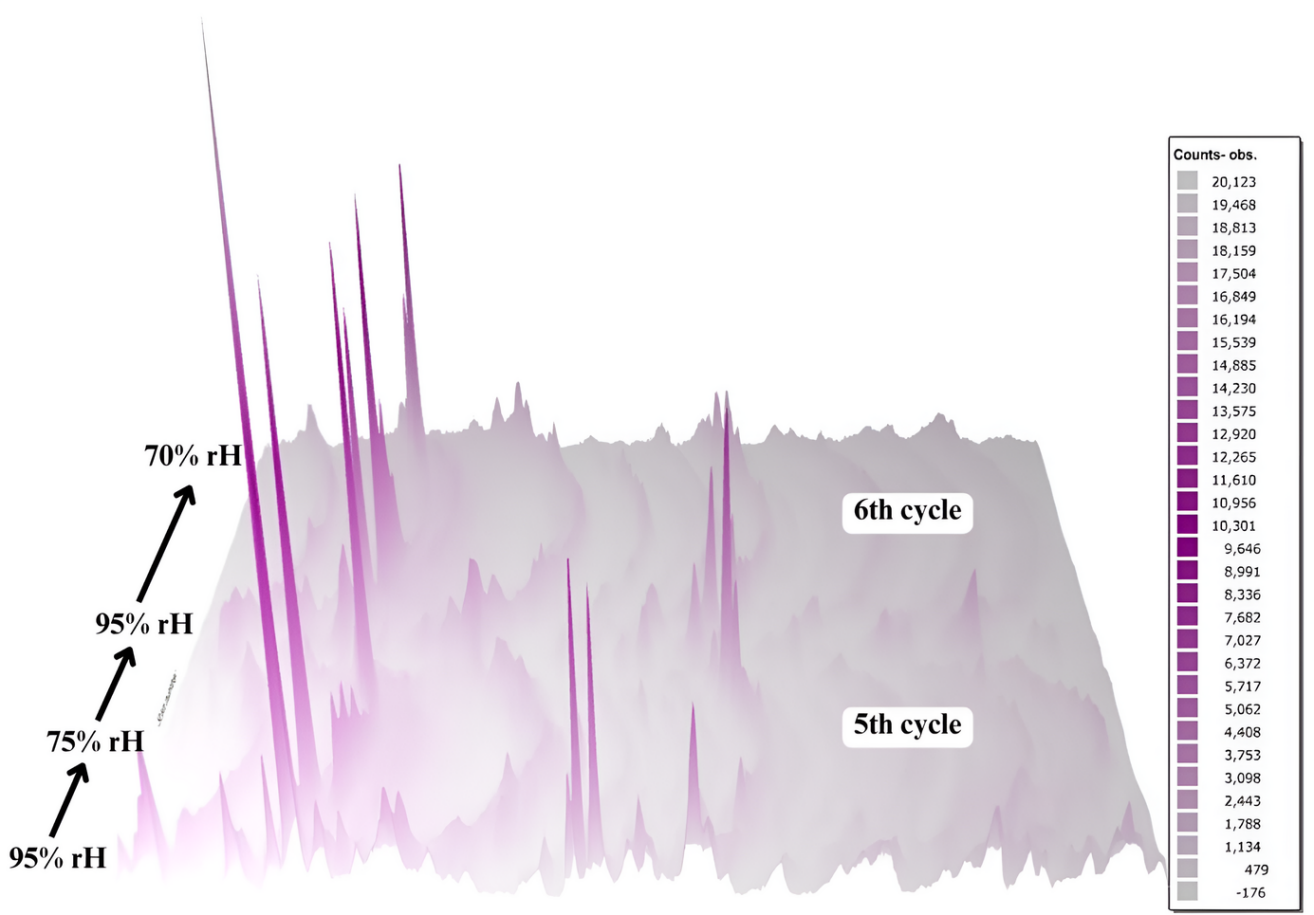
3D surface plot illustrating the 5th and 6th cycles of RH variation of sample mcre2. The plot highlights the evolution of diffraction peak positions and intensities as the sample undergoes alternating dehydration and rehydration.

This pronounced variation in lattice dimensions is clearly evident from the distinct peak shifts in the 3D surface plot of the fifth and sixth cycles, indicating a continuous alteration in diffraction peak positions (Fig. 9 ▸). Upon gradual rehydration of the samples to their original RH values, the unit-cell parameters showed partial restoration. However, the unit cell remained contracted, with a reduced volume of 3.28%.

### 3.3. The effect of humidity variation on HI and *m*-nitro­phenol crystals

**Figure 10:**
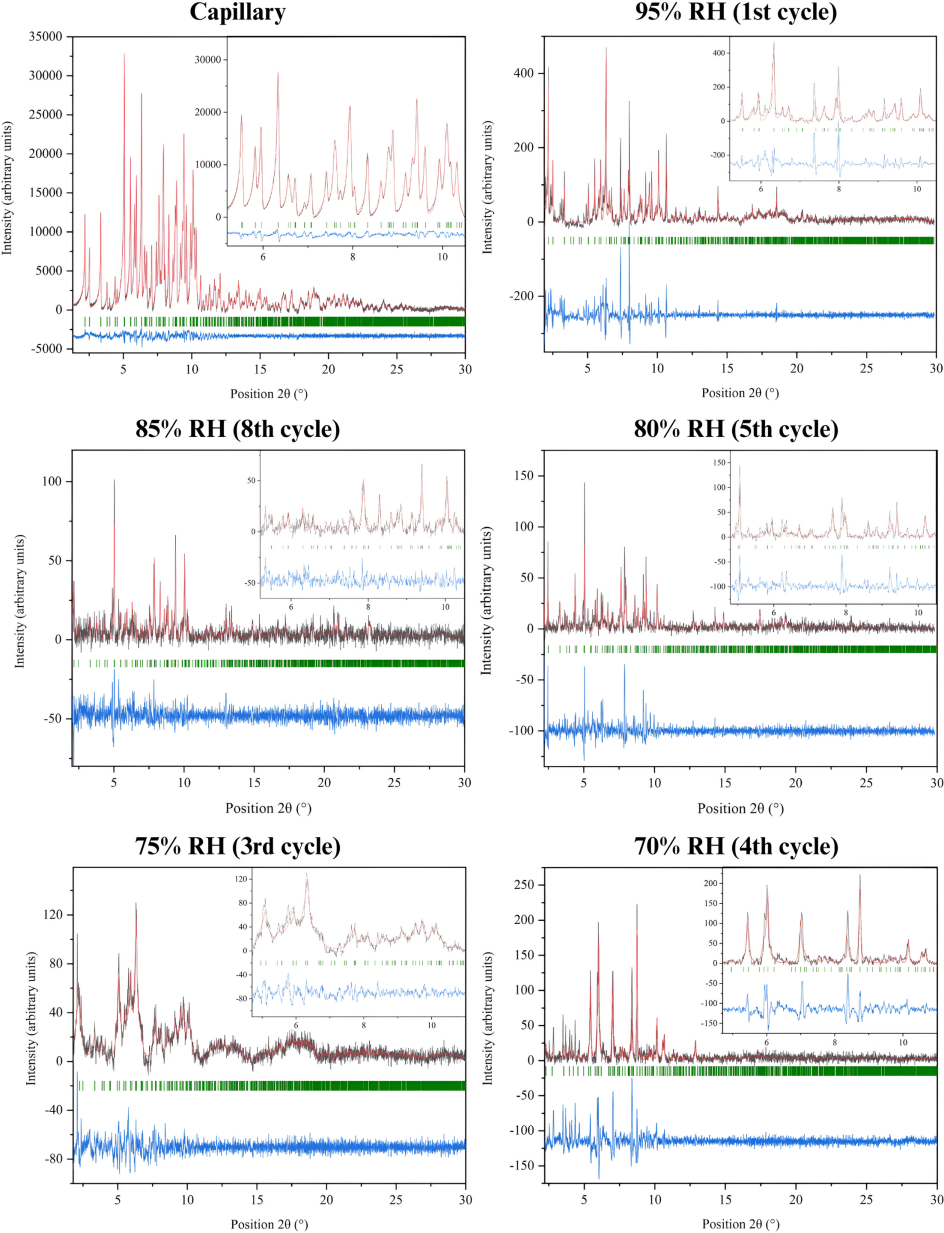
Pawley fits of XRPD data for the HI–*m*-nitro­phenol complex, obtained using the laboratory X’Pert Pro diffractometer under ambient (capillary) and non-ambient conditions. In each plot, the black line represents the experimental data, the red line is the calculated pattern and the blue line shows the difference profile. Green vertical bars correspond to Bragg reflections associated with the rhombohedral space group *R*3. Insets provide a magnified view of the 2θ range from 5° to 11°, highlighting high-intensity Bragg peaks.

Pawley analysis of the XRPD data upon gradual dehydration and rehydration for the mnit1, mnit2, mnit3 and mnit4 samples revealed significant changes in the unit-cell parameters (Fig. 10 ▸; see also the supporting information). A satisfactory diffraction signal was observed within the RH range of 95% to 70% RH. However, at RH levels below 70%, there was a noticeable loss of diffraction signal, indicating a loss of crystallinity, particularly during the last three cycles (*i.e.* the 6th, 7th and 8th cycles). For the mnit3 sample, crystallinity was lost during the 6th cycle at 75% RH, resulting in amorphous patterns for the subsequent steps of the cycle. Notably, crystallinity was restored at 85% RH during rehydration. Despite this recovery, the sample exhibited reduced stability during the 7th cycle, collapsing at RH levels below 90% during dehydration. Similarly, mnit4 crystals collapsed below 70% RH during dehydration but regained structure upon rehydration at 90% RH. These cycles confirmed the reversible behavior of the HI crystal structure, suggesting that the loss of crystallinity due to prolonged dehydration is not permanent and can be restored during rehydration, at least when rehydrated to an RH level of 85%.

Furthermore, the quality of the diffraction data did not show any improvement in terms of the signal-to-noise ratio across the eight cycles. A 60 min interval was sufficient for the sample to equilibrate to the new RH level, as individual scans at each RH level showed no noticeable differences. Any discrepancies would have been apparent if the sample had not fully equilibrated to the new conditions.

In cycles 7 and 8, where the measurements were conducted simultaneously, mnit3 (cycle 7) lost its crystallinity at 90% RH, while mnit4 (cycle 8) lost crystallinity at 70% RH. This difference is probably due to the fact that the mnit3 sample had already undergone two additional dehydration–rehydration cycles (the 5th and 6th cycles) compared with the mnit4 sample, which was measured for the first time. After three cycles of humidity variation, it is likely that the mnit3 sample experienced structural destabilization, leading to the collapse of its crystal structure at higher dehydration levels than the ‘fresh’ mnit4 sample. Radiation damage to mnit3 does not appear to have influenced this observation, as its crystallinity was restored during hydration.

**Figure 11:**
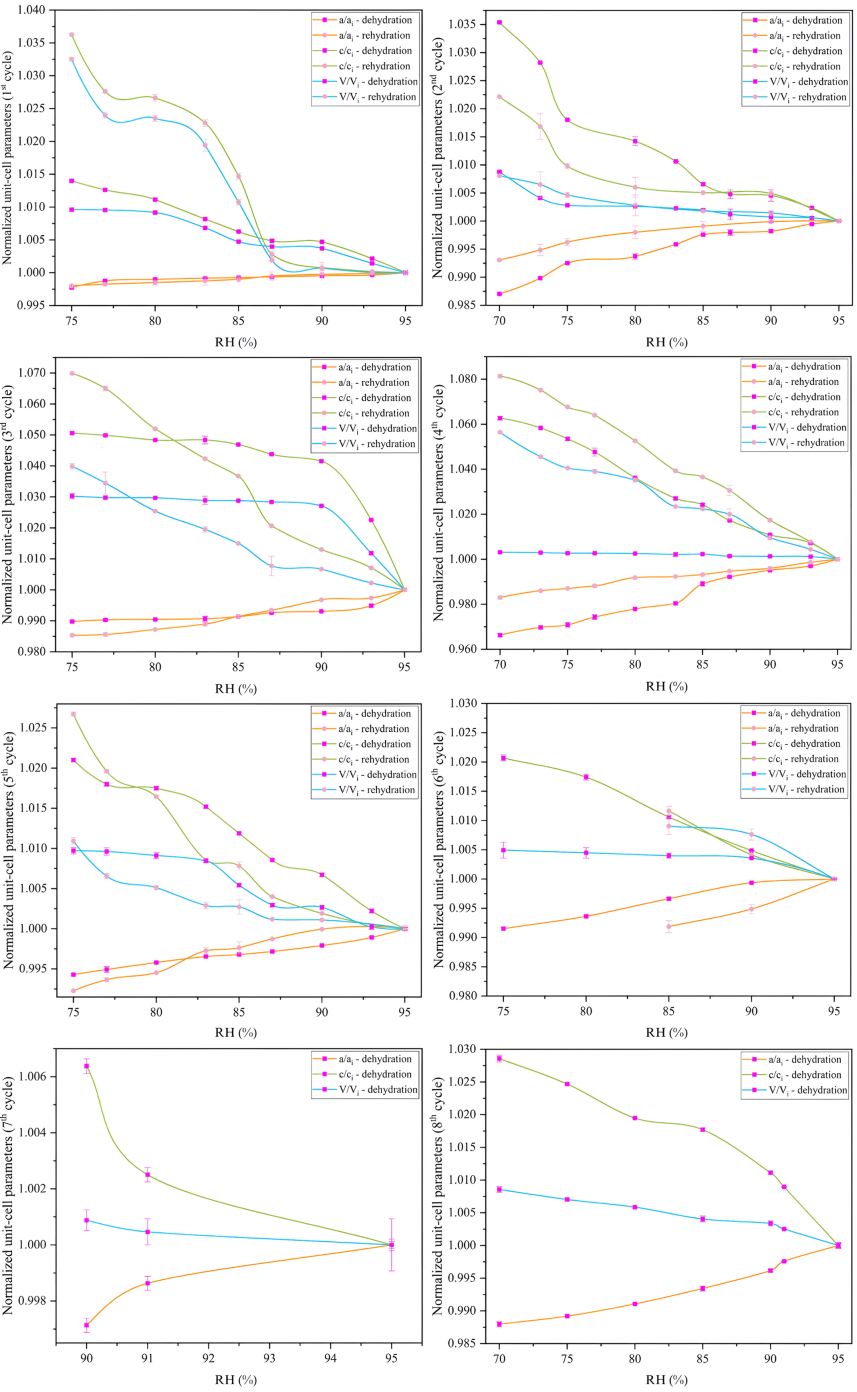
Evolution of the normalized unit-cell parameters *a*/*a*_i_, *c*/*c*_i_ and volume *V*/*V*_i_ as a function of RH for the six dehydration and rehydration cycles of the HI–*m*-nitro­phenol complex. The normalization was calculated relative to the initial unit-cell parameter values at 95% RH for the dehydration and rehydration process of each cycle. The plots highlight the hysteresis in unit-cell parameter variation, with distinct differences observed between dehydration and rehydration phases, indicating the structural response of the crystals to environmental changes.

Dehydration up to 70% resulted in a maximum 3.37% decrease in the *a* and *b* rhombohedral axes, while the *c* axis progressively increased with a maximum variation of 6.27% during the 4th cycle (Table 4 ▸). Apart from the anisotropic variation of the unit-cell parameters (Fig. 11 ▸), the most significant hysteresis is observed along the *c* axis (Fig. 8 ▸). Upon rehydration of the samples, the unit-cell parameters returned with some minor deviations to the initial values, with the unit-cell volume remaining reduced by <1%. However, in cycles 1 and 4, the volume decreased by 2.22% and 5.19%, respectively, compared with the initial values of the first step of each cycle.

## 4. Discussion

Data derived from *in situ* XRPD measurements of HI under controlled RH variation highlight the susceptibility of protein crystals to gradual changes in their physico-chemical environment. Polycrystalline samples of HI complexed with phenolic ligands *m*-cresol and *m*-nitro­phenol underwent significant changes in unit-cell parameters in response to varying RH conditions.

Structural models of the HI complexes provide insights into the binding motifs of the ligands. The rhombohedral structure of the HI–*m*-cresol complex revealed a typical binding pattern in the phenolic pockets of HI, consistent with previous HI complexes involving other phenolic ligands (Karavassili *et al.*, 2020 ▸; Norrman & Schluckebier, 2007 ▸; Smith *et al.*, 2000 ▸; Triandafillidis *et al.*, 2020 ▸). The structure of the HI–*m*-nitro­phenol complex provides the first evidence of this phenolic molecule binding to HI, inducing the R_6_ conformation of the protein. However, in this case, instead of the hydroxyl group of the phenolic molecule forming a hydrogen bond with the amide group of Cys11, an oxygen atom from the nitro group forms this interaction. Additionally, the hydroxyl group of the ligand may form weak non-covalent interactions with surrounding residues, potentially stabilizing its binding within the phenolic pocket. This interaction could explain a stronger binding affinity between the protein and the ligand, influencing the behavior of the complex under fluctuating environmental conditions.

For the HI–*m*-cresol complex, diffraction patterns collected at RH levels ranging from 95% to 70% revealed continuous peak shifts, indicating changes in lattice dimensions, particularly a gradual isotropic decrease in unit-cell parameters and a reduction in unit-cell volume. The most abrupt decrease occurred between 87% and 80% RH, suggesting a critical RH threshold for the sample. In contrast, for the HI–*m*-nitro­phenol complex, the polycrystalline sample exhibited significant changes in unit-cell parameters, but unlike the HI–*m*-cresol complex, the changes were anisotropic upon dehydration. Specifically, the *a* axis decreased while the *c* axis increased, and rehydration led to an increase in the *a* axis and a decrease in the *c* axis. The unit-cell volume, which is primarily affected by the *a* parameter, increased during dehydration and decreased during rehydration. The data suggest that, upon consecutive cycles of dehydration and rehydration, the unit-cell volume of the HI complexes gradually reduced. Notably, the variation in unit-cell parameters and volume became more moderate with each cycle, indicating that RH cycling may lead the crystals to a more stable form with less solvent content, with no phase transition observed in the samples.

This study marks the first attempt to investigate the impact of RH cycling on the stability of crystalline insulin. Prior research has mainly focused on examining physical stability in response to pH changes, phase stability, and thermal stability tests under constant or fluctuating temperatures (Brange & Langkjoer, 1993 ▸; Kaufmann *et al.*, 2021 ▸). Studies on insulin’s thermal stability, particularly in the presence of phenolic ligands such as phenol and *m*-cresol, have shown a decline in stability compared with resorcinol, probably due to the destabilizing effects of some phenolic preservatives (Huus *et al.*, 2006 ▸). In this study, we observed differing effects on insulin stability under RH fluctuations between the *m*-cresol and *m*-nitro­phenol complexes. Notably, the complex with *m*-nitro­phenol exhibited less alteration in the crystal structure, suggesting greater stability compared with the *m*-cresol complex. Additionally, previous research by Flores-Fernández *et al.* (2009 ▸) demonstrated that high humidity absorption by lyophilized insulin results in significant structural changes, including a decrease in α-helix content and an increase in β-sheet content. Future structural investigations based on *in situ* data could provide valuable insights into the precise structural modifications occurring in insulin under varying environmental conditions.

Until now, most studies investigating the effect of the environmental RH on protein crystals of lysozyme and ribonuclease A have relied on the use of saturated salt solutions (Sadasivan *et al.*, 1998 ▸; Biswal *et al.*, 2000 ▸). These experiments revealed variations in unit-cell parameters and space groups under different RH conditions. Structural analyses indicated not only a reduction in solvent content upon dehydration but also conformational changes in the protein molecules. Such changes in quaternary structure are attributed to the loss of water molecules that typically interact with protein molecules, therefore leading to different conformation and/or different crystal packing as in the case of hemoglobin (Kaushal *et al.*, 2008 ▸). In our study, we employed a humidity chamber that allows for precise and controlled RH variation, instead of saturated salts. However, due to the limited resolution of the *in situ* XRPD data, structural refinement of the complexes was not feasible. Future measurements will focus on high-resolution synchrotron data collection under non-ambient conditions, with the goal of elucidating the conformational changes occurring in HI complexes.

The results from the *in situ* XRPD measurements support the hypothesis that RH significantly influences insulin crystallinity. Notably, alterations in the unit-cell parameters were observed under varying RH conditions, with partial loss of diffraction intensity occurring at RH levels below 80%, indicative of partial loss of crystallinity. Upon restoring RH to the initial 95%, a corresponding recovery of unit-cell parameters was observed, indicating the restoration of sample crystallinity. *In situ* XRPD plays a pivotal role in investigating the impact of RH variation, as it provides real-time diffraction data of crystalline samples undergoing structural changes in response to alternating environmental conditions inside the chamber. However, the reliability of the data is affected by the loss of crystallinity during prolonged dehydration to lower RH levels within the chamber. Additionally, continuous dehydration tends to shrink the samples on the sample holders, which poses challenges for determining precise unit-cell parameters during Pawley analysis. This issue can be addressed by adjusting the height of the MHC-trans humidity chamber to ensure optimal alignment of the sample within the X-ray beam. Furthermore, the production of microcrystalline samples plays a crucial role in uniformly distributing the sample in front of the X-ray beam and capturing all possible crystal orientations, which in turn influences the quality of the diffraction data.

Pawley analyses indicate that the R_6_ molecular conformation of HI in both the *m*-cresol and *m*-nitro­phenol complexes remains stable throughout the dehydration–rehydration cycles. The changes observed in unit-cell parameters from the *in situ* XRPD measurements suggest that no transition to other conformations, such as T_6_ or T_3_R*^f^*_3_, occurs. This stability is particularly relevant for the development of new antidiabetic formulations, as the R_6_ configuration of HI is considered more stable from a physico-chemical standpoint (Rahuel-Clermont *et al.*, 1997 ▸). However, to confirm this hypothesis and determine the precise molecular conformation of HI, X-ray absorption near-edge structure spectral analysis at controlled humidity levels is necessary. This technique can determine the geometry of the coordination sphere – whether tetrahedral, octahedral or a combination of the two – providing insights into the stereoregulation of T_6_, R_6_ and T_3_R*^f^*_3_ conformations of HI (Frankaer *et al.*, 2012 ▸).

These structural findings also bear clinical relevance. In diabetic patients, cutaneous adverse reactions at insulin infusion sites are frequently observed during insulin therapy. Several studies have shown that these localized inflammatory responses are mediated by excipients such as phenol and *m*-cresol, commonly included in commercial insulin formulations as preservatives. Using *in vivo* mouse models and *in vitro* cell cultures, researchers have demonstrated that *m*-cresol and phenol can induce cellular and tissue cytotoxicity, along with inflammation at the infusion site (Weber *et al.*, 2014 ▸; Kesserwan *et al.*, 2021 ▸). These observations, when viewed in light of our structural data, underscore the dual role of organic ligands in modulating both the physico-chemical stability and biocompatibility of insulin complexes. While *m*-cresol may support stabilization to some extent, its inflammatory potential raises concerns. In contrast, alternative ligands such as *m*-nitro­phenol, guaiacol, 4-hy­droxy­benzoic acid, thymol and vanillin may offer comparable or superior stabilization with potentially fewer adverse effects. Future studies should further explore the effect of these ligands, not only on protein structure but also on inflammation-related pathways such as reactive oxygen species production and tissue repair. Ultimately, these findings could guide the rational design of insulin formulations that balance structural integrity with reduced immunogenicity and improved patient outcomes.

## 5. Conclusions

This study investigates the effects of RH cycling on the stability of crystalline insulin complexes with *m*-cresol and *m*-nitro­phenol, both exhibiting rhombohedral symmetry in the R_6_ conformation. High-resolution XRPD and *in situ* XRPD data collection methods were employed to monitor changes in unit-cell parameters and crystallinity during controlled RH fluctuations. The results reveal significant modifications in the unit-cell dimensions and crystallinity of both complexes in response to RH variations, without any phase transition occurring.

For the HI–*m*-cresol complex, dehydration led to a continuous reduction in unit-cell parameters and a partial loss of crystallinity, with partial recovery upon rehydration. Notably, the most pronounced changes were observed during the dehydration process, particularly at critical RH thresholds. In contrast, the HI–*m*-nitro­phenol complex exhibited anisotropic changes in unit-cell parameters, demonstrating greater stability under varying RH conditions. These findings suggest that the *m*-nitro­phenol complex is more resistant to environmental fluctuations than the *m*-cresol complex, potentially due to differences in ligand binding and interactions with the protein structure.

The study underscores the critical role of environmental factors, particularly humidity, in influencing the stability and performance of pharmaceutical compounds, especially proteins like insulin. Insulin’s susceptibility to RH fluctuations presents a significant challenge for its long-term stability, especially during storage and handling. The differential stability observed between the two insulin complexes suggests that the choice of ligand can significantly affect insulin’s behavior under environmental stress, with *m*-nitro­phenol providing more robust stabilization compared with *m*-cresol.

Furthermore, the presence of *m*-cresol in insulin formulations has been associated with adverse inflammatory responses at infusion sites in diabetic patients. Studies have shown that excipients like *m*-cresol, commonly used for antimicrobial preservation, can induce cell and tissue cytotoxicity, leading to localized inflammation. These findings highlight the dual importance of ensuring the structural stability of insulin under varying environmental conditions and also considering the broader therapeutic implications, such as minimizing excipient-induced inflammation at infusion sites.

Future structural investigations based on *in situ* experimental data are essential for gaining deeper insights into the structural modifications of insulin complexes under dynamic environmental conditions. Such studies could provide valuable information for improving insulin formulation design and optimizing storage conditions, ultimately enhancing the long-term stability and therapeutic efficacy of insulin-based pharmaceutical products. Additionally, future research should explore the role of other excipients in modulating insulin stability and inflammation, paving the way for the development of safer and more effective insulin therapies.

## Supplementary Material

Supporting information. DOI: 10.1107/S1600576725007484/ei5135sup1.pdf

PDB reference: Rhombohedral crystalline form of human insulin complexed with *m*-nitro­phenol, 9qld

PDB reference: Rhombohedral crystalline form of human insulin complexed with *m*-cresol, 9ibb

## Figures and Tables

**Table 1:** Crystallization conditions for HI–ligand complex formation

	HI–*m*-cresol complex	HI–*m*-nitro­phenol complex
Method	Batch	Batch
Initial concentration of HI	19.07 mg ml^−1^	19.00 mg ml^−1^
Initial concentration of ligand	25% *v*/*v* soluble in ethanol	1 *M* soluble in DMSO
Final composition	13.14 mg ml^−1^ HI, 0.80 m*M* zinc acetate, 0.51% *v*/*vm*-cresol in ethanol, 10.25 m*M* sodium thio­cyanate, 0.4 *M* phosphate mixture (NaH_2_PO_4_ and K_2_HPO_4_)	12.78 mg ml^−1^ HI, 0.77 m*M* zinc acetate, 40.06 m*Mm*-nitro­phenol, 10.09 m*M* sodium thio­cyanate, 0.4 *M* phosphate mixture (NaH_2_PO_4_ and K_2_HPO_4_)
pH	7.50	7.50
Total volume in a 2 ml Eppendorf tube	625 µl (500 µl of protein–ligand mixture + 125 µl PO_4_ buffer)	625 µl (500 µl of protein–ligand mixture + 125 µl PO_4_ buffer)

**Table 2:** Data collection parameters for *in situ* XRPD measurements

Sample	Cycle	Initial RH level (%)	Step (%)	No. of steps	Final RH level (%)	No. of scans/RH	Time interval (min)	Temperature (K)
mcre1	1	95	5	9	75	10	45	294.15
	2	95	5	7	80	6	45	294.15
	3	95	5	9	75	10	45	294.15
	4	95	5	9	75	10	45	294.15
mcre2	5	95	2	16	75	10	90	294.15
	6	95	2	21	70	10	60	294.15
								
mnit1	1	95	2	17	75	10	90	294.15
	2	95	2	16	70	10	90	294.15
mnit2	3	95	2	17	75	10	90	294.15
	4	95	2	21	70	10	90	294.15
mnit3	5	95	2	16	75	10	60	294.15
	6	95	5	19	50	10	60	294.15
	7	95	5	20	50	3	60	294.15
mnit4	8	95	5	20	50	10	60	294.15

**Table 3:** Data processing statistics for SCXRD measurements and structure refinement and validation statistics for HI–ligand complex structures with *m*-cresol and *m*-nitro­phenol (PDB entries 9ibb and 9qld, respectively) Values in parentheses correspond to the high-resolution shell. R.m.s.d. root mean square deviation.

	HI–*m*-cresol	HI–*m*-nitro­phenol
*Data processing statistics*		
Wavelength (Å)	0.8731	0.8731
Resolution range (Å)	39.34–1.84 (2.11–1.84)	39.47–2.55 (39.47–2.55)
Space group	*R*3	*R*3
Unit-cell axis *a*, *c* (Å)	78.684, 39.883	78.932, 39.197
Temperature (K)	100	100
Total reflections	15933 (1003)	5809 (753)
Unique reflections	7990 (503)	2966 (379)
Multiplicity	2.0 (2.0)	2.0 (2.0)
Completeness (%)	99.9 (99.6)	99.4 (100.0)
Mean *I*/σ(*I*)	5.6 (1.7)	15.2 (2.2)
Wilson *B* factor (Å^2^)	36.419	74.56
*R*_merge_ (%)	5.0 (40.8)	3.4 (30.8)
*R*_meas_ (%)	7.1 (57.7)	4.8 (43.5)
CC_1/2_	0.993 (0.627)	0.995 (0.618)

*Refinement statistics*		
Reflections used in refinement	7988 (2666)	2965 (2965)
Reflections used for *R*-free	408 (108)	169 (169)
*R*_work_ (%)	17.79 (26.6)	19.06 (19.04)
*R*_free_ (%)	22.29 (32.3)	24.03 (24.16)
No. non-hydrogen atoms	806	759
No. macromolecules	748	727
No. ligands	27	29
No. solvent	31	3
Protein residues	97	98
R.m.s.d. from ideal (bonds)	0.013	0.003
R.m.s.d. from ideal (angles)	2.22	0.57
Ramachandran favored (%)	100	100
Ramachandran allowed (%)	0	0
Ramachandran outliers (%)	0	0
Rotamer outliers (%)	3.61	0
Clashscore	3.36	4.92
Average *B* factor	46.54	83.73

**Table 4:** Changes in the unit-cell parameters of HI–ligand complexes upon gradual dehydration Delta variations were calculated as Δ(*x*_f_ − *x*_i_)/*x*_i_ % between the initial (*x*_i_) and final (*x*_f_) values.

Sample	Cycle	Δ*a*/*a*_i_ (%)	Δ*c*/*c*_i_ (%)	Δ*V*/*V*_i_ (%)
mcre1	1 (95% → 75%)	−5.49	−11.45	−20.91
	2 (95% → 80%)	−2.89	−3.69	−9.19
	3 (95% → 75%)	−3.47	−9.45	−15.61
	4 (95% → 75%)	−3.94	−13.32	−20.00
mcre2	5 (95% → 75%)	−6.06	−13.16	−23.35
	6 (95% → 70%)	−5.22	−10.35	−19.45
				
mnit1	1 (95% → 75%)	−0.23	1.40	0.96
	2 (95% → 70%)	−1.29	3.54	0.88
mnit2	3 (95% → 75%)	−1.02	5.06	3.02
	4 (95% → 70%)	−3.37	6.27	0.31
mnit3	5 (95% → 75%)	−0.57	2.10	0.97
	6 (95% → 50%)	−0.85†	2.07†	0.49†
	7 (95% → 50%)	−0.29†	0.64†	0.09†
mnit4	8 (95% → 50%)	−1.20†	2.86†	0.86†

† Delta variation values were calculated using the parameter value from the final step of the dehydration process where the sample still retained crystallinity, rather than the lowest RH level of the cycle. For the 6th cycle, this level was 75%; for the 7th, 90%; and for the 8th, 70%.
