# Supplementary material for: Exploring humidity effects on polycrystalline human insulin–ligand complexes: preliminary crystallographic insights
Source: J Appl Crystallogr. 2025 Oct 10;58(Pt 6):1920–35. doi: 10.1107/S1600576725007484 (PMC12810493; doi:10.1107/S1600576725007484)
Supplement: Supplementary file 1 [file j-58-01920-sup1.pdf]

JOURNAL OF APPLIED CRYSTALLOGRAPHY

Volume 58 (2025)

Supporting information for article:

Exploring Humidity Effects on Polycrystalline Human Insulin–Ligand Complexes: Preliminary Crystallographic Insights

Angelos Kontarinis, Christina Papaefthymiou, Stamatina Kafetzi, Marios Konstantopoulos, Dimitris Koutoulas, Max Nanao, Gerd Schluckebier, Mathias Norrman, Natalia Dadivanyan, Detlef Beckers, Thomas Degen, Eleftheria Rosmaraki, Andrew Fitch and Irene Margiolaki

Table S1 Lattice parameters of HI & *m*-cresol polycrystalline sample as extracted via Pawley refinement method using *HighScore Plus* of the *in-situ* XRPD data collected during the first cycle of relative humidity variations.

Data were collected utilizing a laboratory X- ray powder diffractometer (X'Pert Pro) equipped with an MHC trans humidity chamber from Anton Paar (λ =1.540585(3) Å, room temperature).

Cycle	21 °C	RH (%)	Space Group	Unit-cell parameters a (Å)	c (Å)	V (x10^5) (Å3)	χ^2	R_{wp} (%)
1st	dehydration	95	*R3* (R_6)	79.72(2)	40.589(9)	2.2341(7)	9.11387	6.1125
		90	*R3* (R_6)	79.24(2)	39.960(9)	2.1733(8)	6.33525	4.4375
		85	*R3* (R_6)	77.15(9)	38.15(4)	1.966(3)	2.95156	2.5487
		80	*R3* (R_6)	76.26(9)	36.30(4)	1.828(3)	2.99523	2.9609
		75	*R3* (R_6)	75.34(3)	35.94(3)	1.767(2)	2.55682	2.6597
	rehydration	75	*R3* (R_6)	75.34(3)	35.94(3)	1.767(2)	2.55682	2.6597
		80	*R3* (R_6)	75.64(7)	35.99(2)	1.783(2)	2.79042	2.8675
		85	*R3* (R_6)	76.81(9)	36.59(3)	1.870(3)	2.98806	2.848
		90	*R3* (R_6)	78.20(2)	38.72(4)	2.051(3)	4.4033	3.395
		95	*R3* (R_6)	79.52(2)	40.547(8)	2.2208(6)	8.86136	5.0498

Table S2 Lattice parameters of HI & *m*-cresol polycrystalline sample as extracted via Pawley refinement method using *HighScore Plus* of the *in-situ* XRPD data collected during the second cycle of relative humidity variations.

Data were collected utilizing a laboratory X- ray powder diffractometer (X'Pert Pro) equipped with an MHC trans humidity chamber from Anton Paar (λ =1.540585(3) Å, room temperature).

Cycle	21 °C	RH (%)	Space Group	Unit-cell parameters a (Å)	c (Å)	V (x10^5) (Å^3)	χ^2	R_{wp} (%)
2nd	dehydration	95	*R*3 (R_6)	78.95(2)	38.73(1)	2.0911(9)	6.33537	5.2921
		90	*R*3 (R_6)	78.65(8)	38.39(1)	2.056(1)	5.84749	5.841
		85	*R*3 (R_6)	77.98(8)	38.00(5)	2.001(3)	3.7718	3.69536
		80	*R*3 (R_6)	76.67(7)	37.30(4)	1.899(3)	3.9034	3.41105
	rehydration	80	*R*3 (R_6)	76.67(7)	37.30(4)	1.899(3)	3.9034	3.41105
		85	*R*3 (R_6)	77.46(9)	37.86(5)	1.967(3)	4.0884	3.88939
		90	*R*3 (R_6)	78.10(6)	38.18(4)	2.017(2)	5.3476	5.16688
		95	*R*3 (R_6)	78.69(2)	38.48(1)	2.0636(9)	5.52163	4.9922

Table S3 Lattice parameters of HI & *m*-cresol polycrystalline sample as extracted via Pawley refinement method using *HighScore Plus* of the *in-situ* XRPD data collected during the third cycle of relative humidity variations.

Data were collected utilizing a laboratory X- ray powder diffractometer (X'Pert Pro) equipped with an MHC trans humidity chamber from Anton Paar (λ = 1.540585(3) Å, room temperature).

Cycle	21 °C	RH (%)	Space Group	Unit-cell parameters		V (x10^5) (Å^3)	χ^2	R_{wp} (%)
				a (Å)	c (Å)			
3rd	dehydration	95	*R*3 (R_6)	79.06(4)	37.98(2)	2.056(2)	7.54329	5.1138
		90	*R*3 (R_6)	78.69(1)	36.17(1)	1.940(3)	4.41012	3.7894
		85	*R*3 (R_6)	77.35(1)	36.053(6)	1.8682(4)	4.69422	4.4707
		80	*R*3 (R_6)	76.81(2)	35.17(1)	1.7970(8)	4.60327	4.6961
		75	*R*3 (R_6)	76.32(4)	34.39(2)	1.735(1)	4.35111	3.8594
	rehydration	75	*R*3 (R_6)	76.32(4)	34.39(2)	1.735(1)	4.35111	3.8594
		80	*R*3 (R_6)	76.55(6)	36.04(3)	1.829(2)	3.98643	3.8616
		85	*R*3 (R_6)	76.94(6)	37.47(4)	1.921(2)	4.19151	3.7116
		90	*R*3 (R_6)	77.43(6)	37.63(6)	1.954(3)	4.1159	3.6502
		95	*R*3 (R_6)	78.76(3)	38.71(4)	2.079(3)	6.83855	3.9451

Table S4 Lattice parameters of HI & *m*-cresol polycrystalline sample as extracted via Pawley refinement method using *HighScore Plus* of the *in-situ* XRPD data collected during the fourth cycle of relative humidity variations.

Data were collected utilizing a laboratory X- ray powder diffractometer (X'Pert Pro) equipped with an MHC trans humidity chamber from Anton Paar (λ = 1.540585(3) Å, room temperature).

Cycle	21 °C	RH (%)	Space Group	Unit-cell parameters		V (x10^5) (Å^3)	χ^2	R_{wp} (%)
				a (Å)	c (Å)			
4th	dehydration	95	*R*3 (R_6)	79.38(3)	39.03(1)	2.130(1)	7.58604	4.1486
		90	*R*3 (R_6)	78.71(3)	35.87(1)	1.925(1)	5.27576	4.1701
		85	*R*3 (R_6)	77.38(3)	35.077(7)	1.8192(7)	3.52773	3.6644
		80	*R*3 (R_6)	77.16(1)	34.29(1)	1.7686(7)	4.5454	4.7947
		75	*R*3 (R_6)	76.25(2)	33.83(2)	1.7040(9)	3.37110	3.8186
	rehydration	75	*R*3 (R_6)	76.25(2)	33.83(2)	1.7040(9)	3.3711	3.8186
		80	*R*3 (R_6)	76.86(5)	33.93(3)	1.736(2)	3.99812	3.9446
		85	*R*3 (R_6)	76.99(3)	34.33(1)	1.7629(8)	2.94043	3.1925
		90	*R*3 (R_6)	78.65(3)	35.12(1)	1.881(1)	5.8138	4.7363
		95	*R*3 (R_6)	79.20(4)	38.82(2)	2.109(1)	6.92724	4.2276

Table S5 Lattice parameters of HI & *m*-cresol polycrystalline sample as extracted via Pawley refinement method using *HighScore Plus* of the *in-situ* XRPD data collected during the fifth cycle of relative humidity variations.

Data were collected utilizing a laboratory X- ray powder diffractometer (X'Pert Pro) equipped with an MHC trans humidity chamber from Anton Paar (λ = 1.540585(3) Å, room temperature).

Cycle	21 °C	RH (%)	Space Group	Unit-cell parameters a (Å)	Unit-cell parameters c (Å)	V (x10^5) (Å^3)	χ^2	R_{wp} (%)
5th	dehydration	95	$R3$ (R$_6$)	79.797(4)	40.362(2)	2.2257(2)	6.34038	8.3475
		93	$R3$ (R$_6$)	79.74(2)	39.499(9)	2.1753(8)	5.05245	6.8563
		90	$R3$ (R$_6$)	77.84(1)	37.292(3)	1.9571(3)	3.18412	4.6665
		87	$R3$ (R$_6$)	77.53(2)	37.104(7)	1.9317(6)	2.22025	3.3163
		85	$R3$ (R$_6$)	77.35(2)	36.603(9)	1.8970(8)	3.35528	4.8794
		83	$R3$ (R$_6$)	76.55(5)	36.16(2)	1.835(2)	2.32519	3.4727
		80	$R3$ (R$_6$)	76.10(4)	35.91(2)	1.801(1)	2.07834	3.1417
		77	$R3$ (R$_6$)	75.590(7)	35.47(2)	1.755(1)	1.90747	2.9386
		75	$R3$ (R$_6$)	74.96(2)	35.05(2)	1.7060(7)	2.1006	3.2634
	rehydration	75	$R3$ (R$_6$)	74.96(2)	35.05(2)	1.7060(7)	2.1006	3.2634
		77	$R3$ (R$_6$)	75.53(6)	35.33(5)	1.745(3)	2.07585	3.0375
		80	$R3$ (R$_6$)	75.71(2)	35.64(2)	1.7697(7)	2.01879	3.0773
		83	$R3$ (R$_6$)	76.15(3)	36.03(1)	1.8097(8)	3.12632	4.649
		85	$R3$ (R$_6$)	76.81(2)	36.331(5)	1.8565(5)	3.36383	4.8568
		87	$R3$ (R$_6$)	76.94(1)	36.724(4)	1.8828(4)	3.33101	4.8827
		90	$R3$ (R$_6$)	77.57(5)	36.69(3)	1.912(2)	2.54324	3.8524
		95	$R3$ (R$_6$)	79.80(1)	39.033(7)	2.1527(5)	2.82416	5.8158

Table S6 Lattice parameters of HI & *m*-cresol polycrystalline sample as extracted via Pawley refinement method using *HighScore Plus* of the *in-situ* XRPD data collected during the sixth cycle of relative humidity variations.

Data were collected utilizing a laboratory X- ray powder diffractometer (X'Pert Pro) equipped with an MHC trans humidity chamber from Anton Paar (λ = 1.540585(3) Å, room temperature).

Cycle	21 °C	RH (%)	Space Group	Unit-cell parameters a (Å)	Unit-cell parameters c (Å)	V (x10^5) (Å^3)	χ^2	R_{wp} (%)
6th	dehydration	95	$R3$ (R$_6$)	79.923(2)	39.141(3)	2.1652(2)	3.58677	4.96612
		93	$R3$ (R$_6$)	79.647(3)	39.061(6)	2.1459(3)	3.60695	4.67287
		90	$R3$ (R$_6$)	79.372(4)	38.647(6)	2.1086(3)	2.78449	3.86634
		87	$R3$ (R$_6$)	79.143(1)	38.442(5)	2.0853(2)	3.53725	4.74135
		85	$R3$ (R$_6$)	78.218(6)	38.131(7)	2.0204(4)	3.4316	5.02537

		83	*R*3 (R_6)	77.244(7)	37.595(4)	1.9426(2)	2.80138	4.16353
		80	*R*3 (R_6)	76.79(1)	37.08(1)	1.8940(9)	2.41187	3.63671
		77	*R*3 (R_6)	76.26(1)	36.89(1)	1.8587(5)	1.97226	2.99199
		75	*R*3 (R_6)	76.04(1)	36.44(1)	1.8253(8)	1.85308	2.87055
		73	*R*3 (R_6)	75.83(1)	35.87(2)	1.786(1)	1.9126	2.93958
		70	*R*3 (R_6)	75.75(2)	35.09(2)	1.744(1)	1.96641	3.0707
	rehydration	70	*R*3 (R_6)	75.75(2)	35.09(2)	1.744(1)	1.96641	3.0707
		73	*R*3 (R_6)	76.06(1)	35.45(2)	1.776(1)	1.94766	3.04322
		75	*R*3 (R_6)	76.88(2)	35.57(2)	1.821(1)	2.00518	3.08497
		77	*R*3 (R_6)	77.04(1)	35.77(2)	1.838(1)	1.99311	3.09677
		80	*R*3 (R_6)	77.27(2)	35.92(3)	1.8576(1)	1.93032	3.00345
		83	*R*3 (R_6)	77.63(6)	36.13(5)	1.886(3)	2.33216	3.5733
		85	*R*3 (R_6)	77.933(7)	36.446(9)	1.91708(5)	3.05723	4.5994
		87	*R*3 (R_6)	78.297(3)	36.970(7)	1.9628(4)	3.6429	5.23824
		90	*R*3 (R_6)	78.627(4)	37.499(4)	2.0072(2)	2.91075	4.19118
		93	*R*3 (R_6)	78.78(2)	37.62(4)	2.022(2)	2.21916	3.33628
		95	*R*3 (R_6)	78.98(2)	38.07(1)	2.056(1)	2.29444	3.37279

Table S7 Lattice parameters of HI & *m*-nitrophenol polycrystalline sample as extracted via Pawley refinement method using *HighScore Plus* of the *in-situ* XRPD data collected during the first cycle of relative humidity variations.

Data were collected utilizing a laboratory X- ray powder diffractometer (X'Pert Pro) equipped with an MHC trans humidity chamber from Anton Paar (λ = 1.540585(3) Å, room temperature).

Cycle	21 °C	RH (%)	Space Group	Unit-cell parameters		V (x10^5) (Å^3)	χ^2	Rwp (%)
				a (Å)	c (Å)			
1st	dehydration	95	*R*3 (R_6)	80.081(5)	40.317(3)	2.2392(2)	3.57	5.29
		93	*R*3 (R_6)	80.053(6)	40.404(3)	2.2424(2)	5.01	6.99
		90	*R*3 (R_6)	80.044(4)	40.506(4)	2.2475(3)	4.28	6.27
		87	*R*3 (R_6)	80.029(9)	40.513(7)	2.2481(5)	3.90	5.75
		85	*R*3 (R_6)	80.021(6)	40.569(3)	2.2498(2)	2.90	4.20
		83	*R*3 (R_6)	80.014(6)	40.647(4)	2.2545(3)	2.90	4.22
		80	*R*3 (R_6)	80.00(1)	40.766(5)	2.2597(4)	3.54	7.35
		77	*R*3 (R_6)	79.98(1)	40.826(5)	2.2606(4)	4.35	6.72
		75	*R*3 (R_6)	79.90(1)	40.881(6)	2.2607(6)	2.69	4.21
	rehydration	75	*R*3 (R_6)	79.90(1)	40.881(6)	2.2607(6)	2.69	4.21
		77	*R*3 (R_6)	79.92(2)	40.54(1)	2.242(1)	2.46	4.68
		80	*R*3 (R_6)	79.94(3)	40.50(2)	2.241(1)	2.61	4.09
		83	*R*3 (R_6)	79.96(5)	40.35(2)	2.232(2)	2.62	3.78

	85	*R*3 (R_6)	79.98(3)	40.03(2)	2.213(1)	4.15	6.41
	87	*R*3 (R_6)	80.02(5)	39.56(2)	2.1937(2)	3.65	5.15
	90	*R*3 (R_6)	80.04(5)	39.48(2)	2.191(2)	4.13	6.01
	93	*R*3 (R_6)	80.05(1)	39.457(3)	2.1896(3)	4.25	6.19
	95	*R*3 (R_6)	80.06(1)	39.45(1)	2.1895(7)	4.14	5.63

Table S8 Lattice parameters of HI & *m*-nitrophenol polycrystalline sample as extracted via Pawley refinement method using *HighScore Plus* of the *in-situ* XRPD data collected during the second cycle of relative humidity variations.

Data were collected utilizing a laboratory X- ray powder diffractometer (X'Pert Pro) equipped with an MHC trans humidity chamber from Anton Paar (λ = 1.540585(3) Å, room temperature).

Cycle	21 °C	RH (%)	Space Group	Unit-cell parameters		V (x10^5) (Å^3)	χ^2	R_{wp} (%)
				a (Å)	c (Å)			
2nd	dehydration	95	*R*3 (R_6)	79.883(5)	39.291(2)	2.1714(1)	4.13	5.96
		93	*R*3 (R_6)	79.84(1)	39.383(9)	2.1726(6)	4.05	5.88
		90	*R*3 (R_6)	79.74(2)	39.47(4)	2.173(2)	3.54	5.32
		87	*R*3 (R_6)	79.72(4)	39.48(3)	2.174(2)	2.96	5.36
		85	*R*3 (R_6)	79.69(1)	39.549(7)	2.1756(5)	3.97	6.12
		83	*R*3 (R_6)	79.552(8)	39.708(2)	2.1763(2)	1.98	3.66
		80	*R*3 (R_6)	79.38(4)	39.85(3)	2.1771(1)	1.64	3.23
		75	*R*3 (R_6)	79.285(5)	39.999(1)	2.1775(1)	1.55	2.47
		73	*R*3 (R_6)	79.07(1)	40.399(4)	2.1874(4)	1.61	2.11
		70	*R*3 (R_6)	78.849(9)	40.681(4)	2.1904(3)	1.49	2.31
	rehydration	70	*R*3 (R_6)	78.849(9)	40.681(4)	2.1904(3)	1.49	2.31
		73	*R*3 (R_6)	78.99(8)	40.47(9)	2.187(5)	1.18	5.74
		75	*R*3 (R_6)	79.10(5)	40.19(2)	2.183(1)	1.22	3.43
		80	*R*3 (R_6)	79.24(9)	40.04(7)	2.179(4)	1.31	3.24
		85	*R*3 (R_6)	79.326(7)	40.001(1)	2.1768(2)	1.41	3.40
		90	*R*3 (R_6)	79.39(2)	39.996(1)	2.176(1)	1.32	3.68
		95	*R*3 (R_6)	79.399(6)	39.800(2)	2.1729(2)	1.28	6.27

Table S9 Lattice parameters of HI & *m*-nitrophenol polycrystalline sample as extracted via Pawley refinement method using *HighScore Plus* of the *in-situ* XRPD data collected during the third cycle of relative humidity variations.

Data were collected utilizing a laboratory X- ray powder diffractometer (X'Pert Pro) equipped with an MHC trans humidity chamber from Anton Paar (λ = 1.540585(3) Å, room temperature).

Cycle	21 °C	RH (%)	Space Group	Unit-cell parameters		V (x10^5) (Å^3)	χ^2	R_{wp} (%)
				a (Å)	c (Å)			
3rd	dehydration	95	*R3* (R6)	80.269(7)	39.957(2)	2.2296(2)	3.35	8.52
		93	*R3* (R6)	79.86(4)	40.858(8)	2.2560(9)	4.62	10.49
		90	*R3* (R6)	79.712(8)	41.616(7)	2.2900(4)	4.31	10.95
		87	*R3* (R6)	79.675(5)	41.707(2)	2.2929(2)	4.00	9.09
		85	*R3* (R6)	79.574(9)	41.831(3)	2.2938(3)	2.97	7.03
		83	*R3* (R6)	79.52(7)	41.89(5)	2.294(3)	3.77	8.36
		80	*R3* (R6)	79.50(1)	41.899(4)	2.2958(4)	3.71	7.09
		77	*R3* (R6)	79.49(2)	41.95(1)	2.296(1)	2.56	4.15
		75	*R3* (R6)	79.45(2)	41.98(1)	2.297(2)	1.64	2.83
	rehydration	75	*R3* (R6)	79.45(2)	41.98(1)	2.297(2)	1.64	2.83
		77	*R3* (R6)	79.47(5)	41.79(3)	2.285(8)	1.77	3.08
		80	*R3* (R6)	79.600(7)	41.279(3)	2.2651(2)	1.89	3.12
		83	*R3* (R6)	79.74(5)	40.90(2)	2.252(2)	2.00	3.58
		85	*R3* (R6)	79.94(3)	40.68(1)	2.242(4)	3.19	5.52
		87	*R3* (R6)	80.10(1)	40.05(1)	2.2260(7)	1.59	9.16
		90	*R3* (R6)	80.371(8)	39.750(7)	2.2236(4)	4.46	7.99
		93	*R3* (R6)	80.42(1)	39.517(5)	2.2138(4)	4.00	7.51
		95	*R3* (R6)	80.623(4)	39.239(3)	2.2089(2)	5.27	8.43

Table S10 Lattice parameters of HI & *m*-nitrophenol polycrystalline sample as extracted via Pawley refinement method using *HighScore Plus* of the *in-situ* XRPD data collected during the fourth cycle of relative humidity variations.

Data were collected utilizing a laboratory X- ray powder diffractometer (X'Pert Pro) equipped with an MHC trans humidity chamber from Anton Paar (λ = 1.540585(3) Å, room temperature).

Cycle	21 °C	RH (%)	Space Group	Unit-cell parameters		V (x10^5) (Å^3)	χ^2	R_{wp} (%)
				a (Å)	c (Å)			
4th	dehydration	95	*R*3 (R_6)	80.683(9)	39.269(3)	2.2138(3)	4.84	7.83
		93	*R*3 (R_6)	80.44(1)	39.550(3)	2.2163(3)	4.59	8.01
		90	*R*3 (R_6)	80.29(1)	39.695(7)	2.2166(5)	4.70	8.37
		87	*R*3 (R_6)	80.05(2)	39.946(9)	2.2169(8)	4.19	8.14

		85	$R3$ (R_6)	79.80(8)	40.22(2)	2.2188(3)	4.77	9.31
		83	$R3$ (R_6)	79.10(7)	40.33(5)	2.2185(3)	2.57	4.63
		80	$R3$ (R_6)	78.90(3)	40.69(1)	2.2194(1)	2.43	4.36
		77	$R3$ (R_6)	78.61(8)	41.14(7)	2.2197(5)	1.30	7.15
		75	$R3$ (R_6)	78.33(8)	41.37(3)	2.2198(3)	2.51	4.03
		73	$R3$ (R_6)	78.24(6)	41.56(2)	2.2203(2)	1.98	3.16
		70	$R3$ (R_6)	77.96(6)	41.73(3)	2.2206(6)	1.94	3.34
	rehydration	70	$R3$ (R_6)	77.96(6)	41.73(3)	2.2206(6)	1.94	3.34
		73	$R3$ (R_6)	78.20(2)	41.49(1)	2.1977(9)	2.12	3.60
		75	$R3$ (R_6)	78.28(2)	41.20(1)	2.1871(9)	2.02	3.48
		77	$R3$ (R_6)	78.37(6)	41.06(2)	2.184(2)	2.40	4.09
		80	$R3$ (R_6)	78.66(6)	40.62(2)	2.176(2)	2.39	4.05
		83	$R3$ (R_6)	78.699(8)	40.106(3)	2.1512(2)	2.18	3.56
		85	$R3$ (R_6)	78.77(5)	40.00(1)	2.149(1)	2.65	4.42
		87	$R3$ (R_6)	78.89(4)	39.77(9)	2.144(5)	2.98	4.94
		90	$R3$ (R_6)	78.99(6)	39.26(3)	2.122(2)	2.79	5.20
		93	$R3$ (R_6)	79.20(2)	38.886(8)	2.1112(2)	3.51	5.79
		95	$R3$ (R_6)	79.562(9)	38.284(4)	2.0988(3)	3.74	6.27

Table S11 Lattice parameters of HI & *m*-nitrophenol polycrystalline sample as extracted via Pawley refinement method using *HighScore Plus* of the *in-situ* XRPD data collected during the fifth cycle of relative humidity variations.

Data were collected utilizing a laboratory X- ray powder diffractometer (X'Pert Pro) equipped with an MHC trans humidity chamber from Anton Paar (λ = 1.540585(3) Å, room temperature).

Cycle	21 °C	RH (%)	Space Group	Unit-cell parameters		V (x10^5) (Å^3)	χ^2	R_{wp} (%)
				a (Å)	c (Å)			
5th	dehydration	95	$R3$ (R_6)	80.067(8)	39.892(4)	2.2147(4)	2.27	6.38
		93	$R3$ (R_6)	79.98(1)	39.98(1)	2.2152(7)	2.33	7.99
		90	$R3$ (R_6)	79.90(1)	40.160(9)	2.2206(6)	1.76	5.97
		87	$R3$ (R_6)	79.84(1)	40.233(6)	2.2212(5)	1.56	5.22
		85	$R3$ (R_6)	79.81(1)	40.366(7)	2.2267(5)	2.25	6.87
		83	$R3$ (R_6)	79.79(1)	40.498(7)	2.2334(5)	2.45	7.89
		80	$R3$ (R_6)	79.73(1)	40.59(1)	2.2349(9)	2.13	6.93
		77	$R3$ (R_6)	79.66(3)	40.61(1)	2.236(1)	1.84	4.94
		75	$R3$ (R_6)	79.61(1)	40.73(1)	2.2362(9)	1.71	4.89
	rehydration	75	$R3$ (R_6)	79.61(1)	40.73(1)	2.2362(9)	1.71	4.89
		77	$R3$ (R_6)	79.72(2)	40.448(8)	2.2265(7)	1.55	4.30
		80	$R3$ (R_6)	79.79(1)	40.322(7)	2.2233(6)	1.90	5.02

		83	*R*3 (R_6)	80.01(3)	40.01(1)	2.2184(9)	2.39	6.49
		85	*R*3 (R_6)	80.04(6)	39.98(2)	2.218(2)	2.66	7.62
		87	*R*3 (R_6)	80.128(4)	39.829(2)	2.2146(1)	3.23	8.60
		90	*R*3 (R_6)	80.225(9)	39.746(3)	2.2144(3)	2.76	7.07
		95	*R*3 (R_6)	80.23(1)	39.67(1)	2.2120(9)	3.45	8.73

Table S12 Lattice parameters of HI & *m*-nitrophenol polycrystalline sample as extracted via Pawley refinement method using *HighScore Plus* of the *in-situ* XRPD data collected during the sixth cycle of relative humidity variations.

Data were collected utilizing a laboratory X- ray powder diffractometer (X'Pert Pro) equipped with an MHC trans humidity chamber from Anton Paar (λ = 1.540585(3) Å, room temperature).

Cycle	21 °C	RH (%)	Space Group	Unit-cell parameters		V (x10^5) (Å^3)	χ^2	R_{wp} (%)
				a (Å)	c (Å)			
6th	dehydration	95	*R*3 (R_6)	80.231(4)	39.699(1)	2.2131(1)	3.02	7.92
		90	*R*3 (R_6)	80.179(8)	39.892(2)	2.2210(2)	3.11	7.95
		85	*R*3 (R_6)	79.96(2)	40.12(1)	2.2219(9)	2.59	7.31
		80	*R*3 (R_6)	79.72(2)	40.39(7)	2.223(5)	1.27	3.31
		75	*R*3 (R_6)	79.55(2)	40.52(8)	2.224(8)	1.19	3.20
		70	*amorphous*	-	-	-	-	-
		65	*amorphous*	-	-	-	-	-
		60	*amorphous*	-	-	-	-	-
		55	*amorphous*	-	-	-	-	-
		50	*amorphous*	-	-	-	-	-
	rehydration	50	*amorphous*	-	-	-	-	-
		55	*amorphous*	-	-	-	-	-
		60	*amorphous*	-	-	-	-	-
		65	*amorphous*	-	-	-	-	-
		70	*amorphous*	-	-	-	-	-
		75	*amorphous*	-	-	-	-	-
		80	*amorphous*	-	-	-	-	-
		85	*R*3 (R_6)	79.25(8)	39.20(3)	2.132(3)	1.04	3.12
		90	*R*3 (R_6)	79.49(6)	38.91(2)	2.129(2)	1.58	4.25
		95	*R*3 (R_6)	79.90(1)	38.749(8)	2.1129(7)	2.41	6.25

Table S13 Lattice parameters of HI & *m*-nitrophenol polycrystalline sample as extracted via Pawley refinement method using *HighScore Plus* of the *in-situ* XRPD data collected during the seventh cycle of relative humidity variations.

Data were collected utilizing a laboratory X- ray powder diffractometer (X'Pert Pro) equipped with an MHC trans humidity chamber from Anton Paar (λ = 1.540585(3) Å, room temperature).

**Cycle**	**21 °C**	**RH (%)**	**Space Group**	**Unit-cell parameters**		**V (x10⁵) (Å³)**	**χ^2**	***R*wp (%)**
				**a (Å)**	**c (Å)**			
7th	dehydration	95	*R*3 (R_6)	80.24(1)	38.743(8)	2.160(4)	1.99	7.28
		91	*R*3 (R_6)	80.13(2)	38.84(1)	2.161(1)	2.04	7.57
		90	*R*3 (R_6)	80.01(2)	38.99(1)	2.1619(8)	2.07	7.73
		85	*amorphous*	-	-	-	-	-
		80	*amorphous*	-	-		-	-
		75	*amorphous*	-	-	-	-	-
		70	*amorphous*	-	-	-	-	-
		65	*amorphous*	-	-	-	-	-
		60	*amorphous*	-	-	-	-	-
		55	*amorphous*	-	-	-	-	-
		50	*amorphous*	-	-	-	-	-
	rehydration	50	*amorphous*	-		-	-	-
		55	*amorphous*	-	-	-	-	-
		60	*amorphous*	-	-	-	-	-
		70	*amorphous*	-	-	-	-	-
		80	*amorphous*	-	-	-	-	-
		85	*amorphous*	-	-	-	-	-
		90	*amorphous*	-	-	-	-	-
		95	*R*3 (R_6)	79.95(2)	38.84(1)	2.150(1)	1.57	9.05

Table S14 Lattice parameters of HI & *m*-nitrophenol polycrystalline sample as extracted via Pawley refinement method using *HighScore Plus* of the *in-situ* XRPD data collected during the eighth cycle of relative humidity variations.

Data were collected utilizing a laboratory X- ray powder diffractometer (X'Pert Pro) equipped with an MHC trans humidity chamber from Anton Paar (λ = 1.540585(3) Å, room temperature).

**Cycle**	**21 °C**	**RH (%)**	**Space Group**	**Unit-cell parameters**		**V (x10⁵) (Å³)**	**χ^2**	***R*wp (%)**
				**a (Å)**	**c (Å)**			
8th	dehydration	95	*R*3 (R_6)	80.52(3)	39.53(1)	2.220(1)	2.40	5.93
		91	*R*3 (R_6)	80.406(4)	39.805(2)	2.2256(1)	2.48	7.51
		90	*R*3 (R_6)	80.21(2)	39.97(1)	2.2275(9)	2.55	7.09
		85	*R*3 (R_6)	79.99(3)	40.23(1)	2.229(1)	1.51	4.96
		80	*R*3 (R_6)	79.80(1)	40.300(3)	2.2330(3)	2.65	8.07
		75	*R*3 (R_6)	79.65(1)	40.506(6)	2.2356(5)	1.91	6.28
		70	*R*3 (R_6)	79.55(3)	40.66(2)	2.239(1)	1.89	6.70

	65	*amorphous*	-	-	-	-	-
	60	*amorphous*	-	-	-	-	-
	55	*amorphous*	-	-	-	-	-
	50	*amorphous*	-	-	-	-	-
	50	*amorphous*	-	-	-	-	-
	55	*amorphous*	-	-	-	-	-
	60	*amorphous*	-	-	-	-	-
	65	*amorphous*	-	-	-	-	-
rehydration	70	*amorphous*	-	-	-	-	-
	75	*amorphous*	-	-	-	-	-
	80	*amorphous*	-	-	-	-	-
	85	*amorphous*	-	-	-	-	-
	90	*R*3 (R_6)	79.20(1)	39.23(4)	2.151(3)	1.31	3.68
	95	*R*3 (R_6)	79.54(1)	39.219(6)	2.1491(5)	1.51	4.01
